# Purification, characterization and antioxidant activities in vitro of polysaccharides from *Amaranthus hybridus* L.

**DOI:** 10.7717/peerj.9077

**Published:** 2020-04-29

**Authors:** Zizhong Tang, Caixia Zhou, Yi Cai, Yujia Tang, Wenjun Sun, Huipeng Yao, Tianrun Zheng, Hui Chen, Yirong Xiao, Zhi Shan, Tongliang Bu, Xiaoli Wang, Lin Huang, Lin Gou

**Affiliations:** 1 College of Life Sciences, Sichuan Agricultural University, Yaan, China; 2 Sichuan Agricultural University Hospital, Yaan, China; 3 Triticeae Research Institute, Sichuan Agricultural University, Chengdu, China

**Keywords:** *Amaranthus hybridus* L., Physicochemical, Polysaccharides, Antioxidant

## Abstract

**Background:**

*Amaranthus hybridus* L. is an annual, erect or less commonly ascending herb that is a member of the *Amaranthaceae* family. Polysaccharides extracted from traditional Chinese medicines may be effective substances with antioxidant activity.

**Methods:**

In this study, we isolated crude polysaccharides from *A. hybridus* (AHP-M) using microwave-assisted extraction. Then, the AHP-M was purified by chromatography with DEAE-32 cellulose, and two fractions, AHP-M-1 and AHP-M-2, were obtained. The structural characteristics of AHP-M-1 and AHP-M-2 were investigated, and their antioxidant activities were analyzed in vitro.

**Results:**

We found that the monosaccharide composition of AHP-M-1 was different from that of AHP-M-2. The molecular weights of AHP-M-1 and AHP-M-2 were 77.625 kDa and 93.325 kDa, respectively. The results showed that the antioxidant activity of AHP-M-2 was better than that of AHP-M-1. For AHP-M-2, the DPPH radical scavenging rate at a concentration of 2 mg/mL was 78.87%, the hydroxyl radical scavenging rate was 39.34%, the superoxide anion radical scavenging rate was 80.2%, and the reduction ability of Fe^3+^ was approximately 0.90. The total antioxidant capacity per milligram of AHP-M-2 was 6.42, which was higher than that of Vitamin C (Vc).

**Conclusion:**

The in vitro test indicated that AHP-M-1 and AHP-M-2 have good antioxidant activity, demonstrating that *A. hybridus* L. polysaccharide has immense potential as a natural antioxidants.

## Introduction

*Amaranthus hybridus* L. is an annual, erect or less commonly ascending herb that is a member of the *Amaranthaceae* family (Akubugwo et al., 2007; Das, 2016). This plant is often used as a vegetable to treat intestinal bleeding, diarrhea and excessive menstruation (Olusola & Anslem, 2010). The *Amaranthus* genus has a worldwide distribution and harbors between 50 and 70 species (Stetter & Schmid, 2017). There are many varieties of this plant, and it has been reported that many other *Amaranth* species are natural hybrids derived from *A. hybridus* L. (Trucco et al., 2005). *A. hybridus* L. was initially a native plant in North, Central and South America but is naturalized in many other places with warm climates (Prajitha & Thoppil, 2018). In China, the plant grows mainly in the south, including Sichuan, Hunan, Jiangsu and Guizhou (Zhang et al., 2010). The plant is located in a variety of places, including mining wastelands, tailings, barrens and other disturbed habitats (Santiago-Saenz et al., 2018). Recent studies have demonstrated that *Amaranth*, when used as a vegetable, has antioxidant capacity and contains many bioactive components, such as L-ascorbic acid, beta-carotene, polyphenol, anthocyanins and lutein (Das, 2016).

It has been found that free radicals play a key role in the degenerative or pathological processes of various diseases, such as cancer, coronary heart disease, atherosclerosis, neurodegenerative disorders, aging, cataracts and various forms of inflammation (Smith et al., 1996; Dhalla, Temsah & Netticadan, 2000; Jacob & Sotoudeh, 2002; Capek, Machová & Turjan, 2009). Numerous crude extracts and pure natural compounds from plants have been reported to have antioxidant and radical scavenging abilities (Tian et al., 2011). Polysaccharides are widely distributed in animals, plants, and microorganisms, and they are significant biocatalysts and information molecules in organisms (Chen et al., 2015b; Shang et al., 2017). Polysaccharides extracted from traditional Chinese medicines may have antioxidant effects. Polysaccharides with different sources vary in their physicochemical characteristics, such as chemical composition, molecular weight and glycosidic linkages (Chen, Wu & Huang, 2013). Some studies have indicated that polysaccharides play an important role in the prevention of oxidative damage in living organisms (Pang, Chen & Zhou, 2000; Tsiapali et al., 2001) and act as dietary free radical scavengers. Consequently, searching for active natural compounds with possible antioxidant or radical scavenger properties is still of great importance.

*A. hybridus* L. has recently attracted considerable interest because it contains essential diet components, such as protein, vitamins, iron, calcium, and other nutrients that are usually in short supply (Teasdale & Pillai, 2005; Oluwatosin et al., 2009). *A. hybridus* L. has been shown to contain a large amount of squalene, a compound that has both health and industrial benefits (Arunachalam et al., 2016). It has also been reported that this compound has important beneficial effects against cancers (Rao, 1998) and reduces cholesterol levels in the blood (Smith, 2000). However, there is relatively little information related to the isolation, purification and activity determination of polysaccharides from *A. hybridus* L. by microwave-assisted extraction (MAE). Moreover, the antioxidant effects of *A. hybridus* L. polysaccharides have not been characterized. Since structure and functions are closely related, an in-depth study of the structure of these polysaccharides would be of interest (Jahanbin et al., 2011). Therefore, the objective of this study was to focus on the isolation and structural characterization of a water-soluble polysaccharide, AHP-M, from *A. hybridus* L. and to determine its antioxidant activities in vitro.

## Materials and Methods

### Plant material

*A. hybridus* L. was collected from Sichuan Agricultural University in September 2015. Briefly, the plants were dried; afterwards, the samples were stored at room temperature in hermetically sealed black plastic bags under darkness to avoid possible oxidation of the compounds. Finally, the samples were milled into powder using a laboratory grinding machine.

### Chemical reagents

2, 2-Diphenyl-1-picrylhydrazyl (DPPH, HPLC) was purchased from Yuanye Biotechnology Co. (Shanghai, P. R. China). Series standards of dextran, potassium bromide (KBr, SP), and nitrotetrazolium blue chloride (NBT, AR) were purchased from Sigma Chemical Co. (St. Louis, MO, USA). Vitamin C (Vc, AR) was purchased from Sinopharm Chemical Reagent Co. (Shanghai, P. R. China). Fetal bovine serum was purchased from Yuanye Biotechnology Co. (Hangzhou, P. R. China). Other chemicals and solvents used in this study were analytical grade.

### Preparation of crude polysaccharides from *A. hybridus* L.

The extraction of AHP-M was conducted by following the method of Jin with slight modifications (Jin et al., 2019). First, *A. hybridus* L. powder was added to preheated petroleum ether at 70 °C; the mixture was refluxed, and the colorless petroleum ether was extracted to remove surface fats and pigments. Then, the sample was treated with acetone and ethanol to further remove interference components, including free sugars, amino acids and polyphenols. The *A. hybridus* L. extract powder was dried and placed in a dry bottle for use. Then, the abovementioned pretreated *A. hybridus* L. powder (20 g) was extracted using MAE under optimized conditions (a ratio of water to material of 31 mL/g, microwave power of 210 W, and microwave time of 47 min). The filtrate was concentrated to 1/4 volume under reduced pressure and precipitated with 95% ethanol (1:4, v/v) at 4 °C for 24 h. The precipitate was collected by centrifugation (5,000 rpm, 15 min) and deproteinated by Sevag reagent (1-butanol/chloroform, v/v = 1:4). The deproteinization solution was reprecipitated in a four-fold volume of 95% ethanol. Finally, the precipitate was collected and lyophilized to give crude *A. hybridus* L. polysaccharides (AHP-M).

### Scanning electron microscopy analysis

The morphology of AHP-M was observed by scanning electron microscopy (SEM) using a JSM-7500. The AHP-M powder was subjected to a conductive treatment, and then a gold film was plated on the surface. Finally, the microstructure of AHP-M was examined under a high vacuum at a voltage of 15.0 kV, and images were collected at 3,000×*g* and 1,000×*g* magnifications.

### Purification of AHP-M

The AHP-M was dissolved in water to prepare a 10 mg/mL solution, filtered through a 0.45 µm membrane and loaded onto a DEAE-32 cellulose column (Li et al., 2006). The column was eluted with double distilled water and 0.1–1 M NaCl solution at a flow rate of 0.6 mL/min. Sample collection was performed by an automatic collector with a time interval of 10 min. The elution curve was prepared by determining the total sugar content. Single peak components were combined and isolated by concentrating and drying. The main polysaccharide fraction was collected to obtain white purified polysaccharides, named AHP-M-1 and AHP-M-2, which were used for further study.

### Determination of neutral sugar, protein and uronic acid content

The neutral sugar contents in AHP-M, AHP-M-1 and AHP-M-2 were determined by the phenol-sulfuric acid method (Cuesta et al., 2003; Albalasmeh, Berhe & Ghezzehei, 2013) with glucose as the standard. The protein content was determined by the Bradford method (Bradford, 1976) using bovine serum albumin as the standard. The content of alduronic acid was determined by the sulfuric acid-carbazole method (Blumenkrantz & Asboe-Hansen, 1973) with D-glucuronic acid as the standard.

### Fourier transform infrared spectral analysis

The dried AHP-M, AHP-M-1 and AHP-M-2 samples were mixed with spectroscopic-grade potassium bromide powder and then ground and pressed into pellets for Fourier transform infrared (FT-IR) measurements. The FT-IR analysis was performed in a frequency range of 4,000–400 cm^−1^ by an FT-IR instrument (SHIMADZU, 8400 S).

### Ultraviolet spectrometry (UVS) analysis

The dried AHP-M, AHP-M-1 and AHP-M-2 samples were dissolved in water to prepare aqueous polysaccharide solutions, and full-wavelength scans were performed from 200 nm to 800 nm to detect whether impurities such as proteins were present.

### Determination of molecular weight

The molecular weights of AHP-M-1 and AHP-M-2 were determined by gel column chromatography with a Sephadex G-100. The column was calibrated with T-series dextran (T-7, 10, 40, 50 and 500) as the standard, and the molecular weights of the polysaccharides were estimated by reference to the elution standard curve.

### Analysis of monosaccharide composition

The monosaccharide compositions of AHP-M, AHP-M-1 and AHP-M-2 were analyzed by gas chromatography-mass spectrometry (GC-MS). A quartz capillary column (RTX5mx, 30 × 0.25 mm, 0.25 μm) was used with the following parameters: 1.9 mL/min velocity, 1 μL sample quantity, and 40 °C initial temperature. After 2 min, the sample was heated to 290 °C at 6 °C/min and kept for 10 min. The temperature of the detector was 290 °C.

### In vitro antioxidant activity assay

#### DPPH radical scavenging assay

The DPPH radical scavenging activities of AHP-M-1 and AHP-M-2 were investigated using a previous method with a slight modification (Zhang et al., 2015a). Vc was used as the standard antioxidant. AHP-M-1 and AHP-M-2 were separately dissolved in distilled water to prepare polysaccharide solutions of different concentrations (0.4, 0.8, 1.2, 1.6 and 2.0 mg/mL). Two milliliters of DPPH solution was mixed with different concentrations of polysaccharide solution. The mixture was shaken vigorously and then incubated in a dark place at room temperature for 30 min. Finally, the absorbance was measured at 517 nm by a spectrophotometer. The DPPH radical scavenging activity (%) was calculated by the following equation: scavenging rate (%) = [1 − (A_1_ – A_2_)/A_3_] × 100, where A_1_ is the A_517_ of the sample with DPPH, A_2_ is the A_517_ of the sample without DPPH, and A_3_ is the A_517_ of DPPH without the sample.

#### Hydroxyl radical (•OH) scavenging assay

The hydroxyl radical scavenging activities of AHP-M-1 and AHP-M-2 were determined by the method of Liu (Liu & Ng, 2000), and Vc was used as the standard antioxidant. AHP-M-1 and AHP-M-2 were separately dissolved in distilled water to prepare polysaccharide solutions of different concentrations (0.4, 0.8, 1.2, 1.6, and 2.0 mg/mL). A 0.5 mL sample of each concentration was mixed with 0.5 mL of O-phenanthroline (0.75 mM), 1.0 mL of phosphate buffer solution (PBS, 0.15 M, pH 7.4), 0.5 mL of ferrous sulfate (0.75 mM) and 0.5 mL of H_2_O_2_ solution (0.01%). Then, the mixtures were incubated at 37 °C for 30 min. Finally, the absorbance was measured at 510 nm by a spectrophotometer. The •OH radical scavenging activity (%) was calculated by the following equation: scavenging rate (%) = [(A_1_ − A_2_)/(A_3_ − A_2_)] × 100, where A_1_ is the absorbance of the sample after reaction with hydroxyl radicals, A_2_ is the absorbance of the sample, and A_3_ is the absorbance without H_2_O_2_.

#### Superoxide radical (•O^2−^) scavenging assay

The superoxide radical scavenging activities of AHP-M-1 and AHP-M-2 were determined by the Beauchamp and Jia methods (Beauchamp & Fridovich, 1971; Jia, Tang & Wu, 1999). Similarly, Vc was used as the standard antioxidant. AHP-M-1 and AHP-M-2 were separately dissolved in distilled water to prepare polysaccharide solutions of different concentrations (0.4, 0.8, 1.2, 1.6 and 2.0 mg/mL). A 1.0 mL sample of each concentration was mixed with 2.0 mL of PBS (0.05 M, pH 7.4), 2.0 mL of riboflavin (3.3 × 10^−6^ M), 2.0 mL of methionine (0.01 M) and 2.0 mL of NBT (4.6 × 10^−5^ M). Then, the mixtures were illuminated for 30 min. Finally, the absorbance was measured at 560 nm using a spectrophotometer. The •O^2−^ radical scavenging activity (%) was calculated by the following equation: scavenging rate (%) = [ 1 − (A_1_/A_2_)] × 100, where A_1_ is the absorbance of the sample and A_2_ is the absorbance of the control.

#### Measurement of the reducing power

The reducing powers of AHP-M-1 and AHP-M-2 were measured by the Prussian blue method (Hu, 2010). AHP-M-1 and AHP-M-2 were separately dissolved in distilled water to prepare polysaccharide solutions of different concentrations (0.4, 0.8, 1.2, 1.6 and 2.0 mg/mL). A 2.5 mL sample of each concentration was mixed with 2.5 mL of PBS (0.2 M, pH 6.6) and 1 ml of 1% potassium ferricyanide (K_3_ Fe (CN)_6_). The mixture was allowed to incubate at 50 °C for 20 min. Afterwards, the mixture was kept at room temperature in the dark. Then, 2.5 mL of trichloroacetic acid solution (TCA, 10%, w/v) was added, and the solution was centrifuged at 3,000 rpm for 10 min. The upper layer of the solution (2.0 mL) was mixed with 2.0 mL distilled water and FeCl_3_ (0.5 mL, 0.1%), and the absorbance was measured at 700 nm. The absorbance value was defined as the reducing power. At the same time, Vc was analyzed for comparison.

#### Total radical scavenging assay

The total antioxidant activity can well explain the antioxidant capacity of a sample when it acts on the body. The total radical scavenging activities of AHP-M-1 and AHP-M-2 were determined with a total antioxidant capacity (T-AOC) assay kit. One unit of T-AOC was defined as the amount required to increase the absorbance of the reaction system by 0.01 per milligram of sample.

## Results

### Physicochemical properties of AHP-M

#### Analysis of the chemical composition of AHP-M

Table 1 shows the chemical composition of the AHP-M sample. The neutral sugar content was 28.8 ± 1.36%, the alduronic acid content was 23.28 ± 0.20%, and the soluble protein content was 3.77 ± 0.42%.

**Table 1:** The content of neutral sugar, uronic acid and protein in the crude polysaccharide.

Polysaccharide sample	Neutral sugar (%)	Alduronic acid (%)	Soluble protein (%)
AHP-M	28.8 ± 1.36	23.28 ± 0.20	3.77 ± 0.42

**Note:**

AHP-M is Microwave-assisted extract polysaccharide.

#### FT-IR analysis

**Figure 1:**
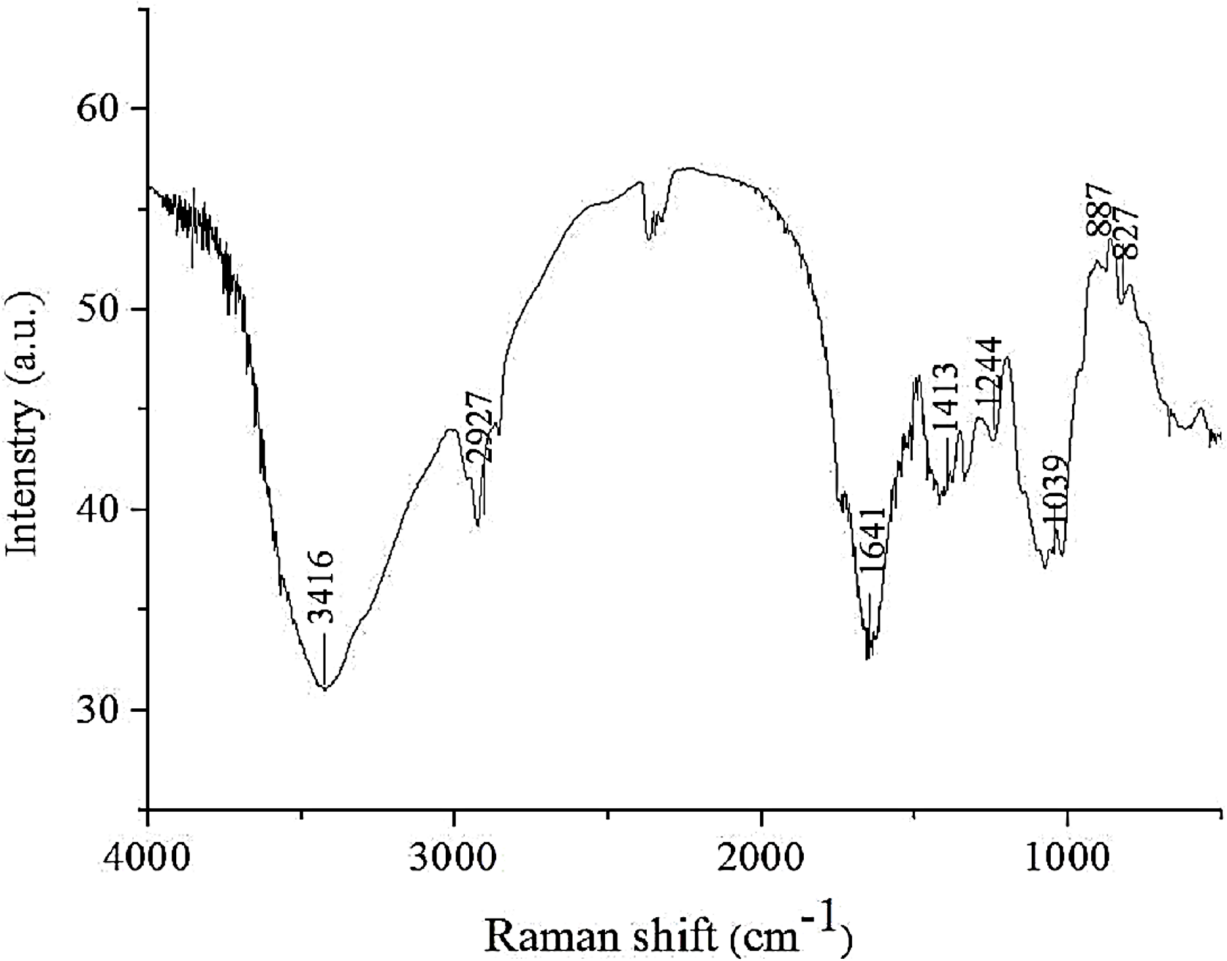
FT-IR spectrum of the polysaccharide AHP-M.

FT-IR spectroscopy is a reliable technique for the analysis of polysaccharide structures, such as glucosidic bonds and functional groups (including organic functional groups). As shown in Fig. 1, the IR spectrum of AHP-M revealed a strong absorption peak at approximately 3,417 cm^−1^ corresponding to O–H stretching vibrations and a weak band at 2,900–2,950 cm^−1^ corresponding to C–H stretching vibrations, indicating the characteristic absorptions of polysaccharides. The absorption peak at 1,638 cm^−1^ was the asymmetric stretching vibration band of C=O, and the absorption peak at 1,413–1,550 cm^−1^ was the stretching vibration band of N–H, which might be related to the protein content in AHP-M. The absorption at 1,039 cm^−1^ indicated a pyranose form of sugar. Moreover, the bands at 827 cm^−1^ and 887 cm^−1^ indicated the coexistence of α- and β-configurations of the sugar units in AHP-M (Pawar & Lalitha, 2014).

#### UVS analysis

**Figure 2:**
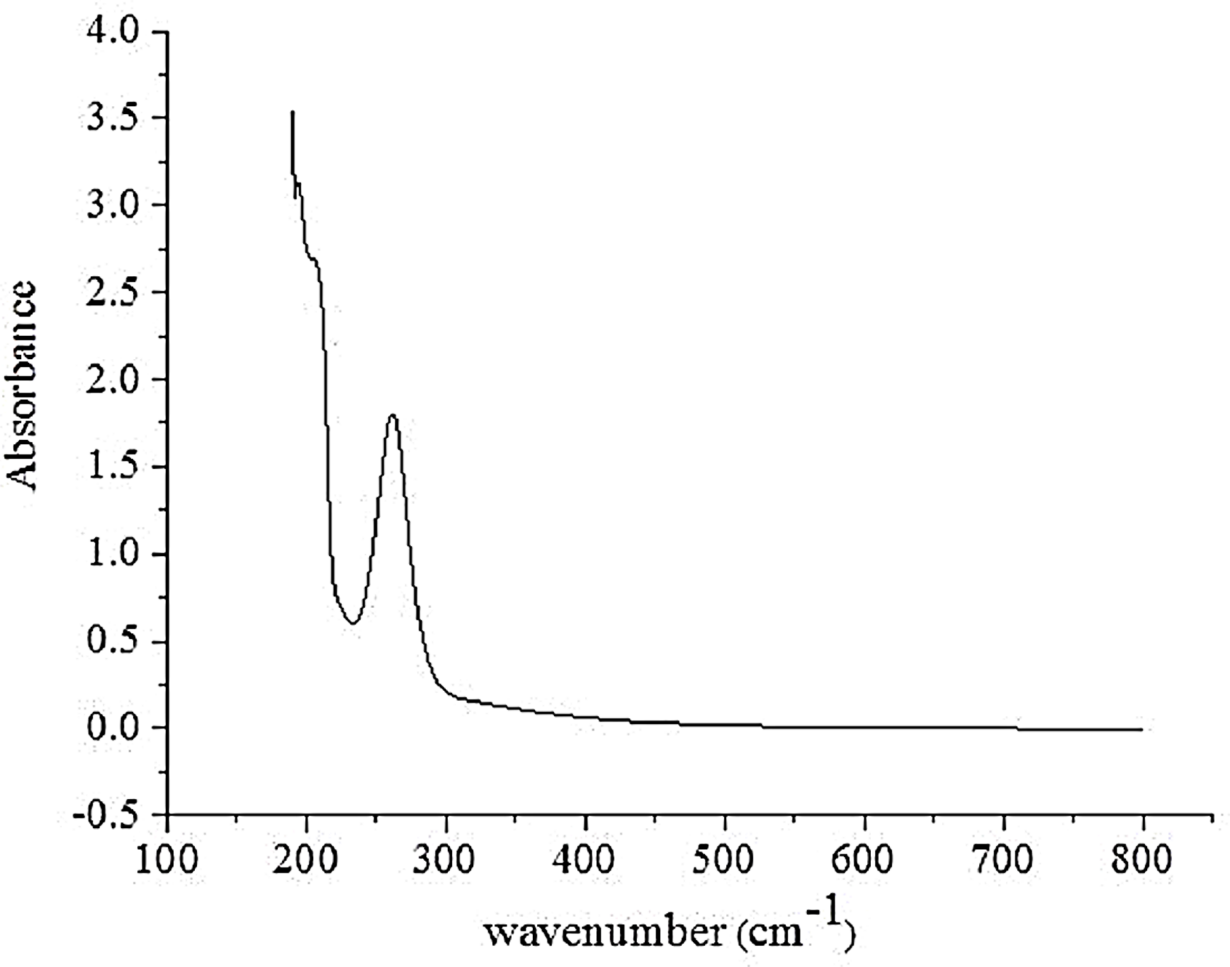
The UVS absorption spectrum of AHP-M.

The UV absorption spectra of the polysaccharides are shown in Fig. 2. There was a strong peak at 260–280 nm, indicating that the obtained AHP-M contained protein and nucleic acid, which is consistent with the results of FT-IR and chemical composition analysis. In addition, no absorption was detected in the UV spectrum at 620 nm, indicating the absence of pigments.

#### SEM analysis

**Figure 3:**
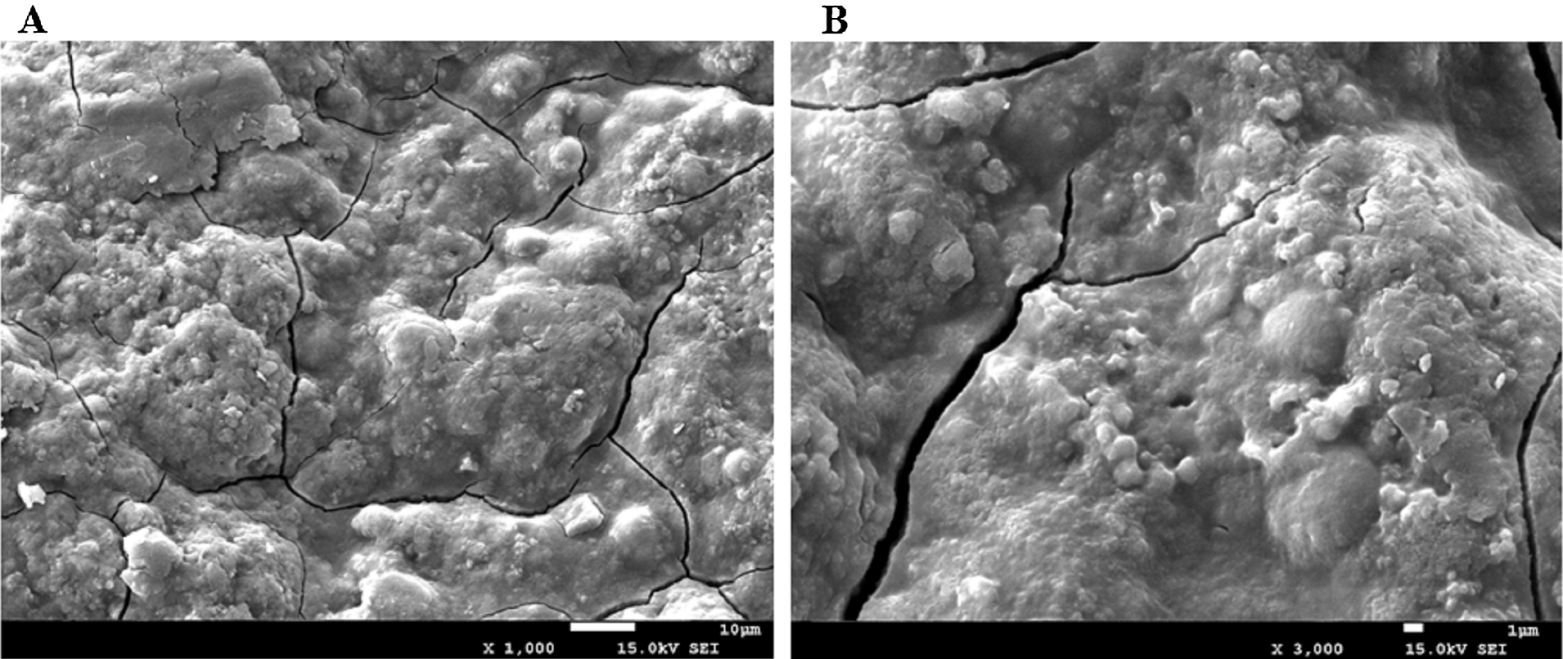
The scanning electron micrographs of AHP-M. (A) AHP-M (1,000×); (B) AHP-M (3,000×).

It has been reported that the shape and structure of polysaccharides may be affected by extraction and purification methods (Ma et al., 2016; Chen et al., 2015a). The effect of the MAE method on the surface of the AHP-M structure was shown in Fig. 3. The surface of the polysaccharide structure was irregular and had some bubble-like structures, which was related to the microwave processing method. The SEM of AHP-M results showed that the surface structure of polysaccharides was affected by microwave-assisted extraction.

### Purification and physicochemical properties of AHP-M

#### Purification of AHP-M

**Figure 4:**
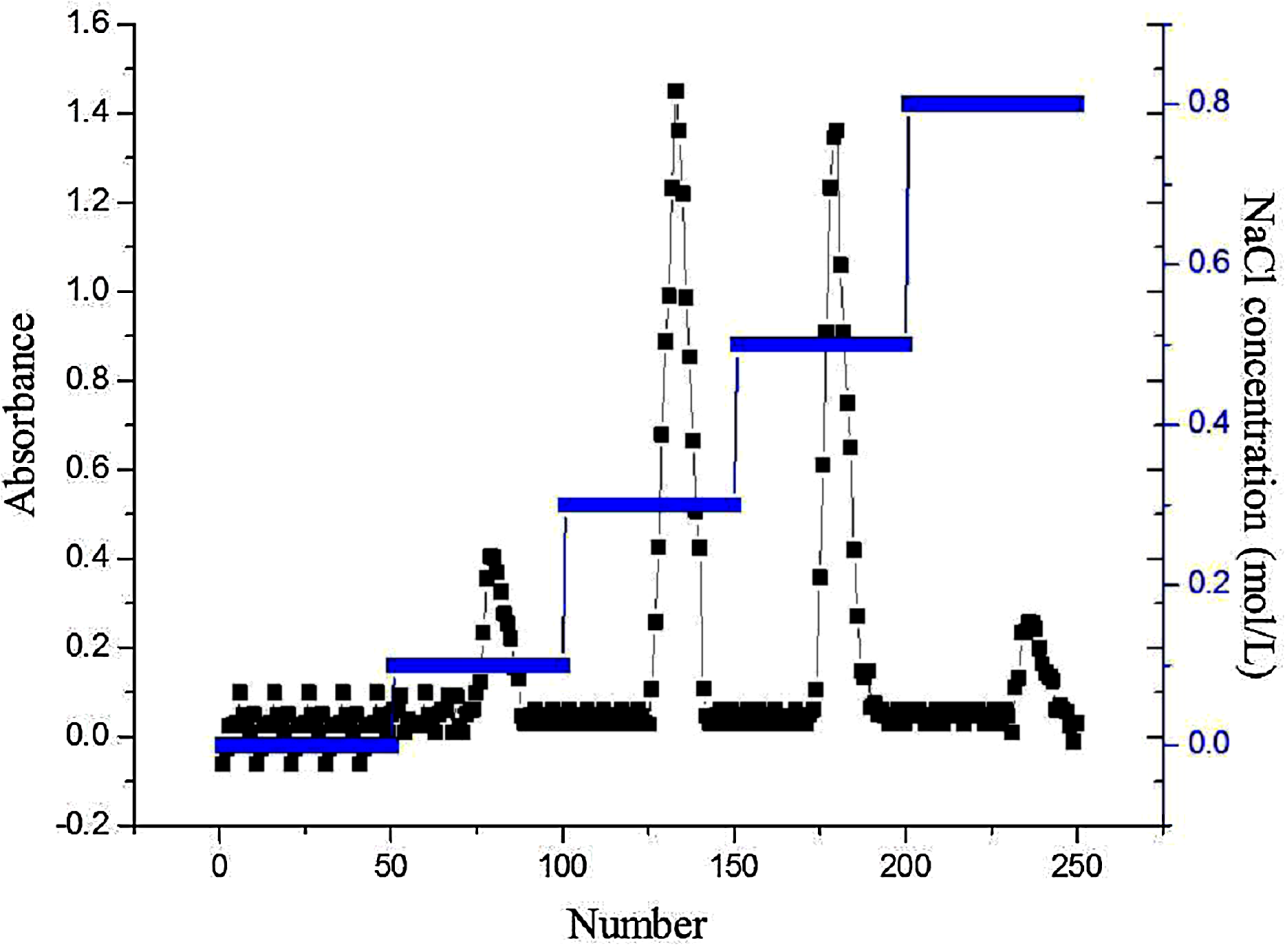
DEAE cellulose-32 column chromatogram of polysaccharide AHP-M.

The crude polysaccharide was purified by DEAE-32 cellulose column chromatography. The elution curve of AHP-M is shown in Fig. 4. There were four peaks in the curve, and no peaks appeared in the 1–50 double distilled water tube elution. Therefore, acidic polysaccharides were the major component of AHP-M, and the sample did not contain neutral polysaccharides. Two main fractions, AHP-M-1 and AHP-M-2, were eluted with 0.3 and 0.5 M NaCl solution, respectively, indicating that they were acidic polysaccharides. Subsequently, the components were collected, and the AHP-M samples were collected through a dialysis membrane (8–14 kDa) for desalination and vacuum drying. The two purified polysaccharide samples were isolated.

#### Chemical composition of purified polysaccharides

The contents of neutral sugar, alduronic acid and soluble protein in the purified polysaccharides are shown in Table 2. The neutral sugar content of AHP-M-1 was 59.9%, which was higher than that of AHP-M-2, and the soluble protein content was lower than that of AHP-M-2. This result indicated that AHP-M-1 was purer than AHP-M-2. In addition, the contents of neutral sugar and alduronic acid in AHP-M-1 and AHP-M-2 were significantly higher than those in the crude polysaccharide, and the protein content was significantly decreased, indicating that the proteins in AHP-M-1 and AHP-M-2 were basically removed and the purification effect was good.

**Table 2:** Contents of neutral sugar, uronic acid and protein in the crude polysaccharide.

AHP sample	Neutral sugar (%)	Alduronic acid (%)	Soluble protein (%)
AHP-M-1	59.9 ± 1.28	32.38 ± 0.42	0.25 ± 0.08
AHP-M-2	50.8 ± 0.87	40.13 ± 0.77	0.64 ± 0.23

#### FT-IR analysis

**Figure 5:**
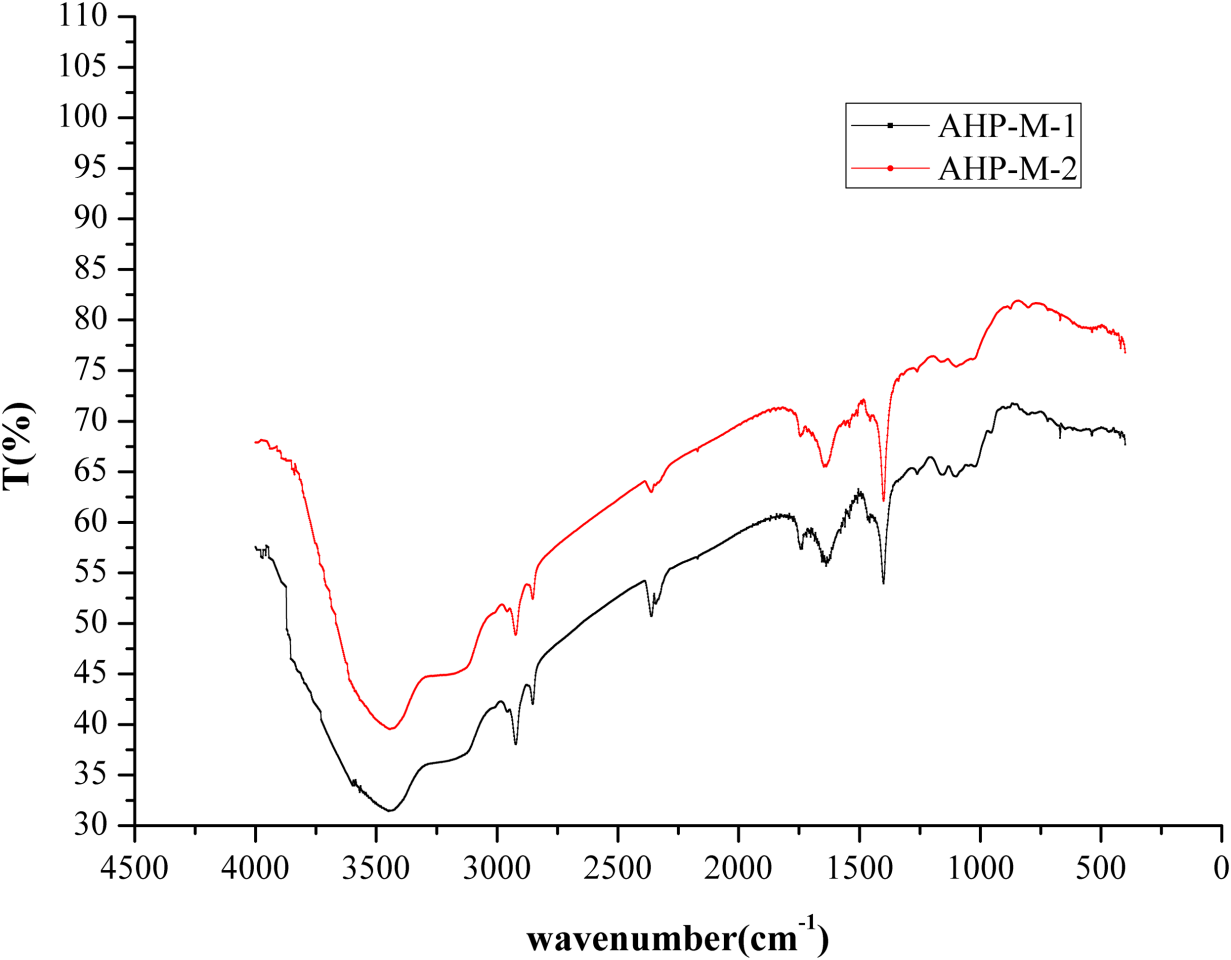
FT-IR spectra of AHP-M-1 and AHP-M-2.

The infrared spectra of AHP-M-1 and AHP-M-2 ranged from 4,000 cm^−1^ to 400 cm^−1^ and are shown in Fig. 5. All of the AHP-M samples had similar FT-IR absorption bands, indicating similarities in their structural features. Each sample showed a strong absorption peak at approximately 3,417 cm^−1^ corresponding to O–H stretching vibrations and a weak band at 2,900–2,950 cm^−1^ corresponding to C-H stretching vibrations. The absorption peak at 1,600 cm^−1^ represented the characteristic stretching vibration of C–O bonds, and the absorption peak at 1,500 cm^−1^ was the stretching vibration band of N–H. The two polysaccharides had absorption peaks at 1,500–1,600 cm^−1^, indicating that they still contained some protein, which was consistent with the chemical composition analysis.

#### UVS analysis

**Figure 6:**
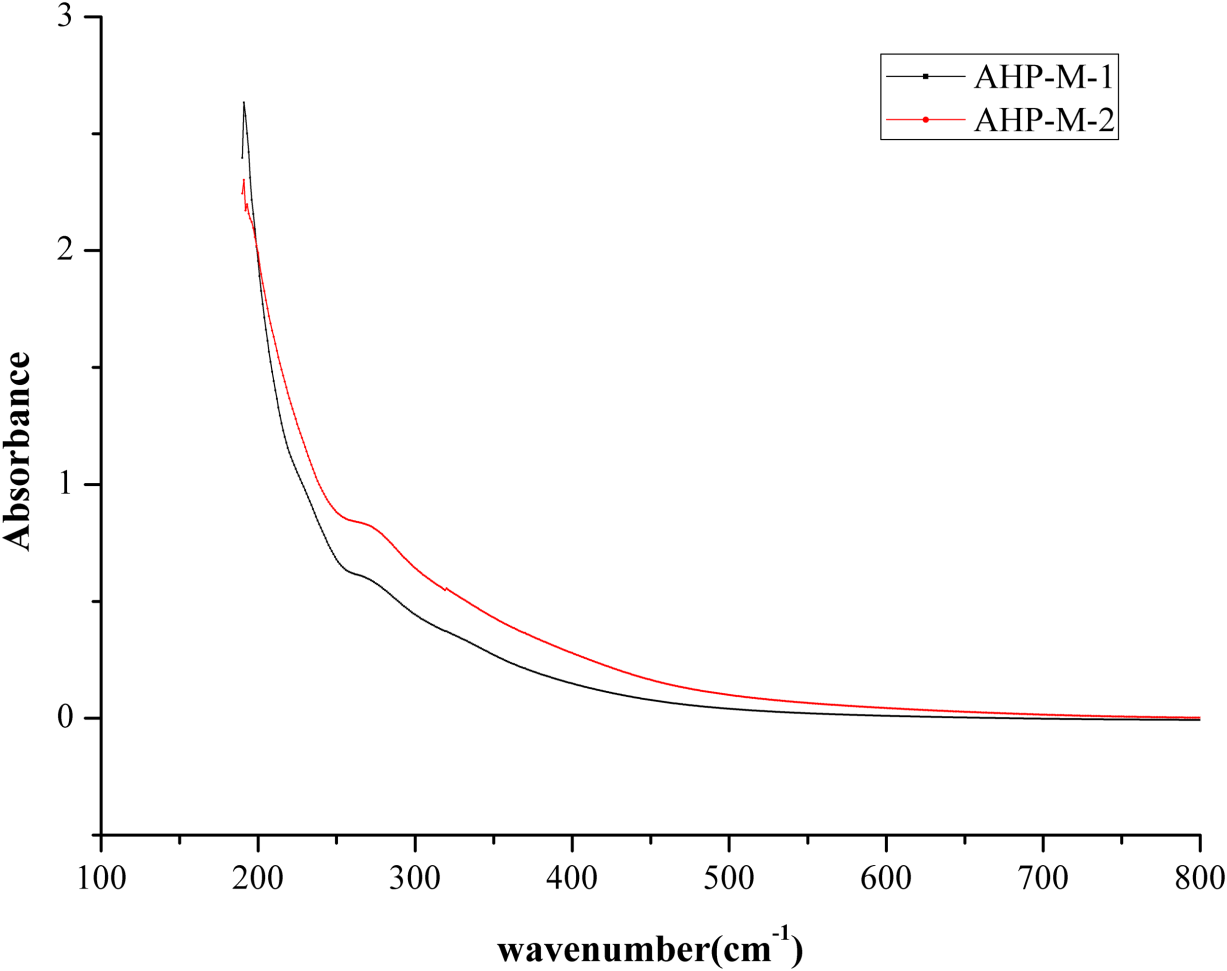
The UVS absorption spectra of AHP-M-1 and AHP-M-2.

The UV spectra of AHP-M-1 and AHP-M-2 are shown in Fig. 6. As seen from the figure, there was no significant difference between the UVS absorption spectra of AHP-M-1 and AHP-M-2. The UVS results showed no absorption at 260 nm but a weak absorption peak at 280. This result implied that there was a small amount of protein in AHP-M-1 and AHP-M-2 but nucleic acid and coloring materials were absent in these polysaccharides. The results were basically consistent with the chemical composition analysis and FT-IR results.

#### Determination of molecular weight

**Figure 7:**
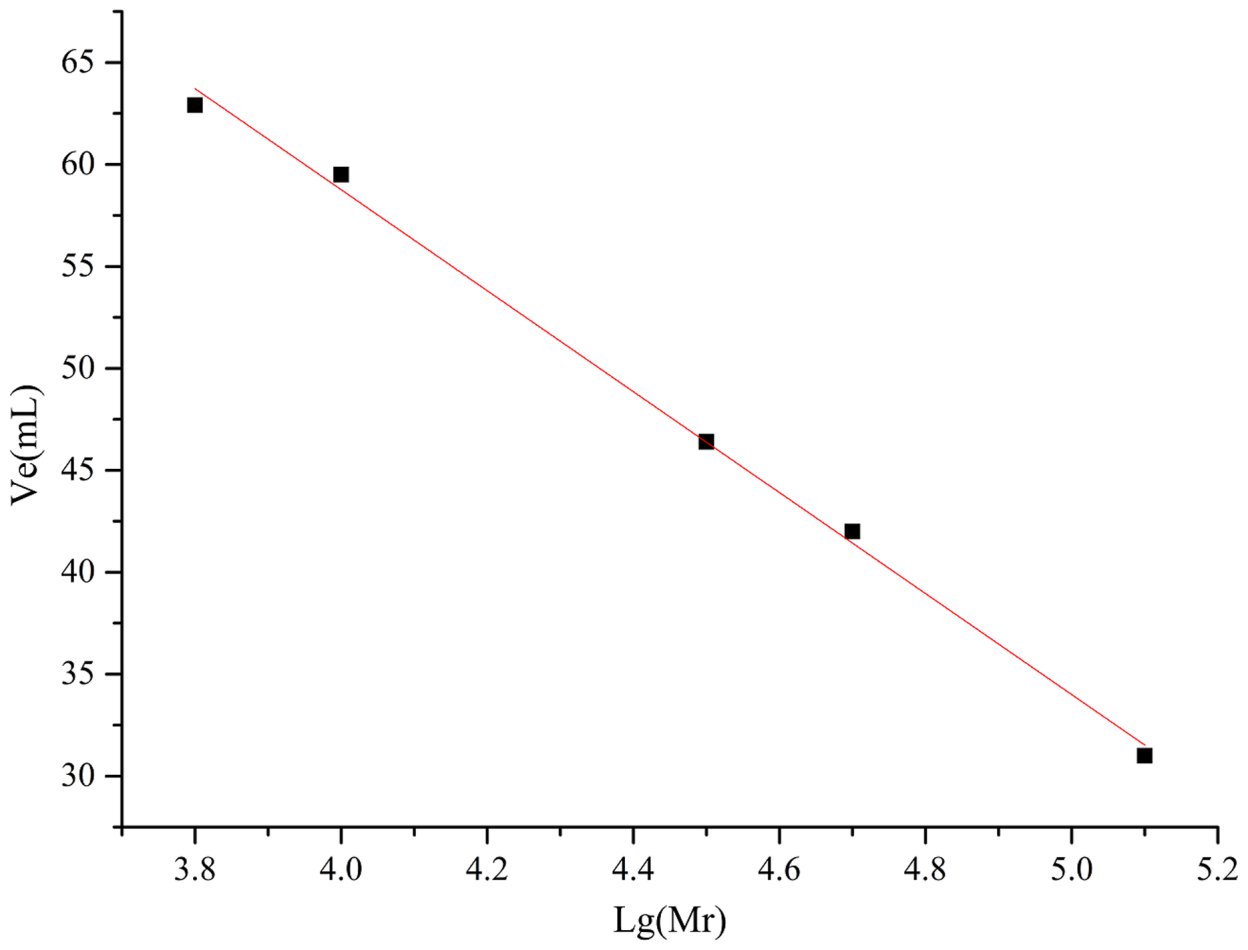
Standard curve of the relative molecular weight.

The molecular weights of AHP-M-1 and AHP-M-2 were determined using a Sephadex G-100 gel filtration column. Ve was plotted as the ordinate, lg (Mr) was plotted as the abscissa of the standard curve, and the formula was Ve = −24.763 lg (Mr) + 158.09, *R*^2^ = 0.9913, as shown in Fig. 7 and Table 3. The molecular weights of AHP-M-1 and AHP-M-2 were estimated to be 77.625 kDa and 93.325 kDa, respectively. The molecular weight of AHP-M-1 was lower than that of AHP-M-2.

**Table 3:** The table of molecular weight.

Polysaccharide sample	AHP-M-1	AHP-M-2
Ve (mL)	37	35
Lg (Mr)	4.89	4.97
Mr (Da)	77,625	93,325

#### Monosaccharide composition

**Figure 8:**
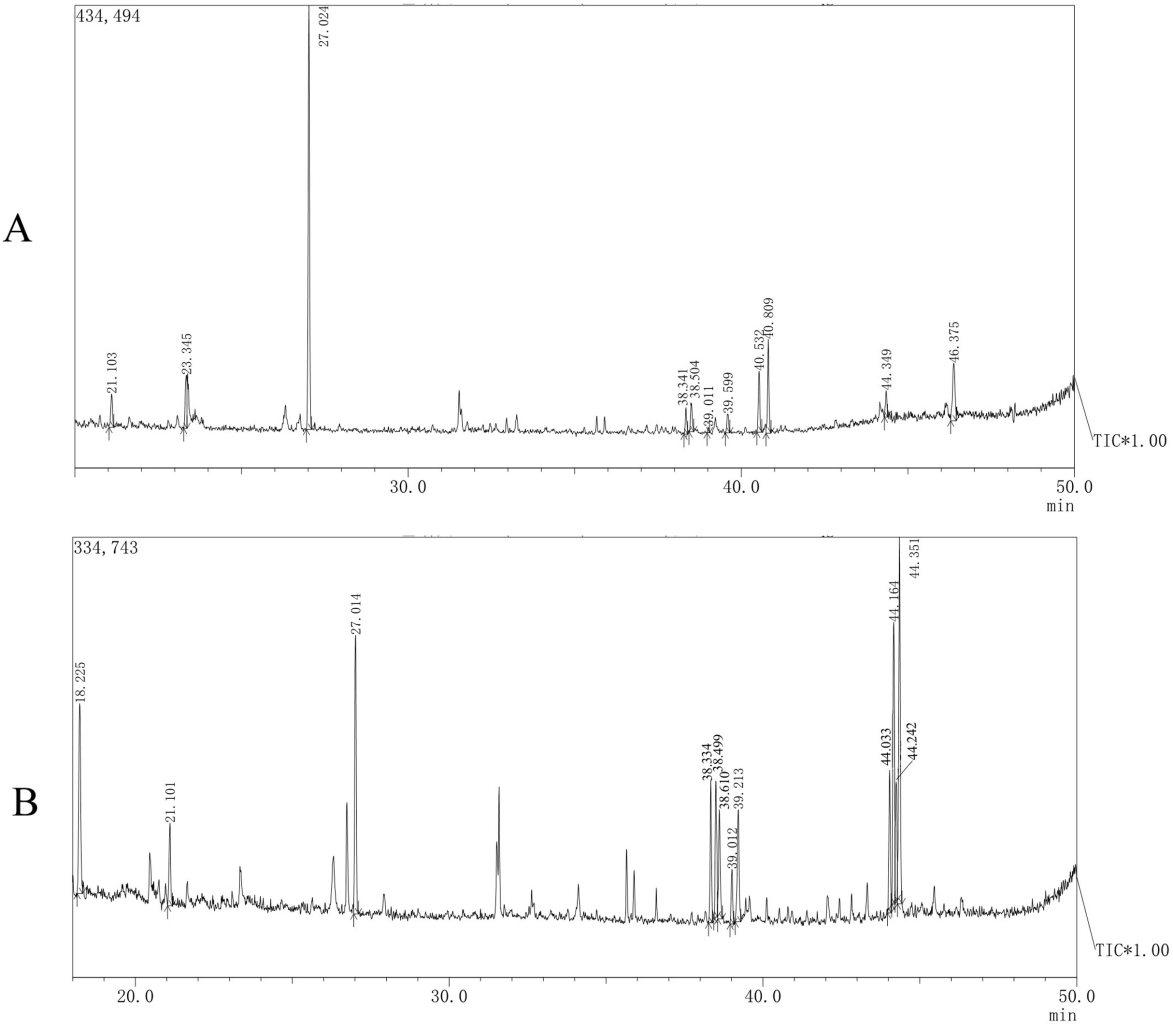
The GC-MS spectrometry of purified polysaccharides of AHP-M-1 (A) and AHP-M-2 (B).

The monosaccharide compositions of AHP-M-1 and AHP-M-2 are shown in Fig. 8 and Table 4. The monosaccharide composition analysis indicated that AHP-M-1 was a heteropolysaccharide and consisted of mannose, rhamnose, and glucose. The presence of mannose (approximately 59.84%) as the main monosaccharide indicated that mannose could be the main backbone of AHP-M-1. Four monosaccharides were found in AHP-M-2: mannose, rhamnose, galactose and glucose; galactose and glucose (approximately 42.29%) exhibited a high content. Therefore, the analyses showed that AHP-M-1 was rich in mannose, whereas AHP-M-2 was rich in glucose. The difference might be related to the raw material and the purification process. It has also been reported that differences in the components of tea polysaccharides were due to different tea raw materials or purification processes (Nie & Xie, 2011).

**Table 4:** The monosaccharide composition of purified polysaccharides (%).

Name	Mannose	Rhamnose	Galactose	Lyxose	Xylose	Fructose	Glucose
AHP-M-1	59.84	17.13	nd	nd	nd	nd	23.03
AHP-M-2	6.69	5.02	35.11	nd	nd	nd	42.29

**Note:**

nd, undetected

### In vitro antioxidant activity

#### DPPH radical scavenging assay

**Figure 9:**
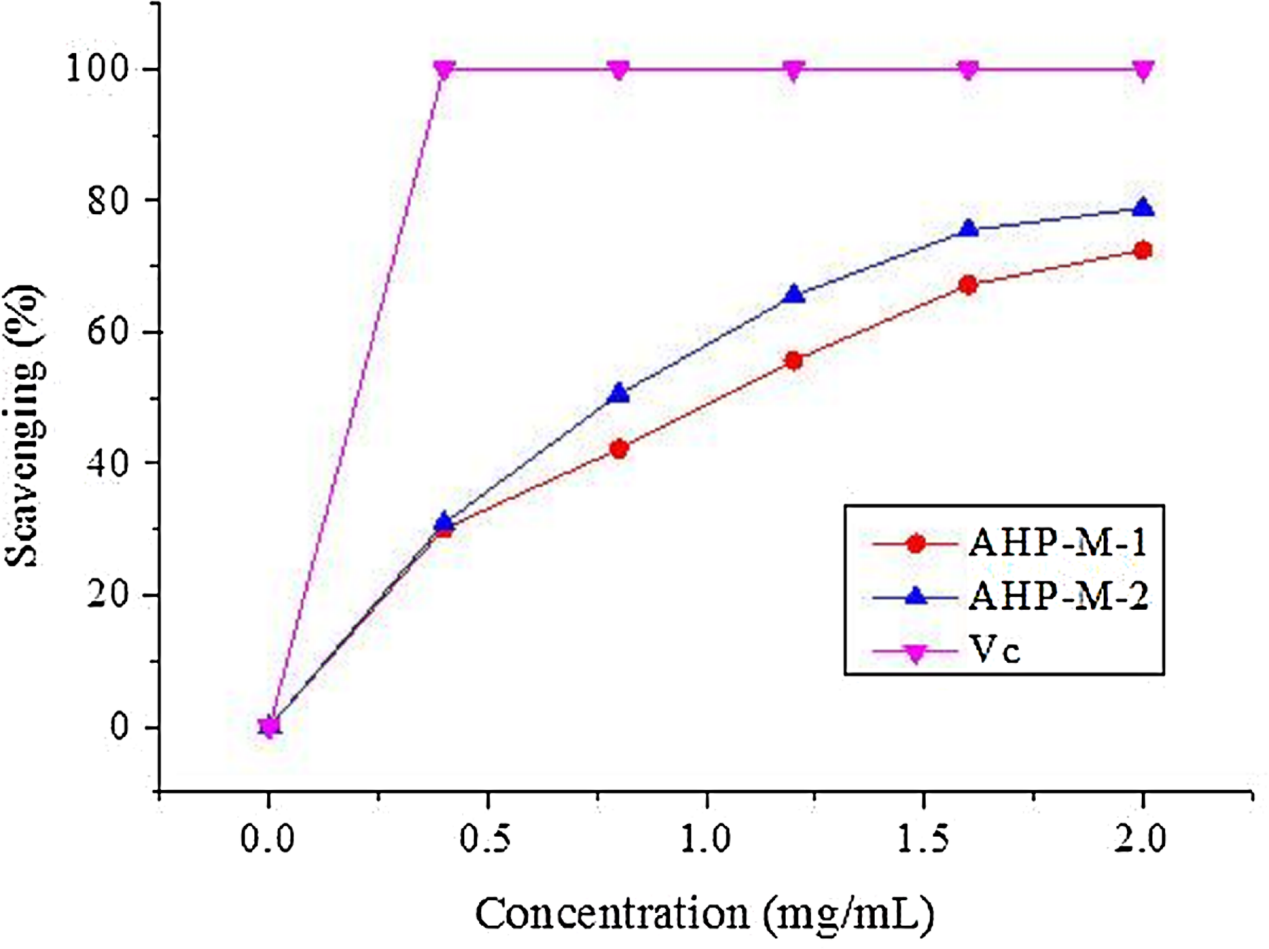
The DPPH radical scavenging ability of the purified polysaccharides.

DPPH is a stable free radical that has been widely accepted as a tool for estimating the free radical scavenging activities of antioxidants (Khled Khoudja, Boulekbache-Makhlouf & Madani, 2014). The scavenging effects of the two purified samples, AHP-M-1 and AHP-M-2, on DPPH radical are shown in Fig. 9. The scavenging effects of AHP-M-1 and AHP-M-2 on DPPH radical increased in a concentration-dependent manner and were 72.41% and 78.9% at 2.0 mg/mL, respectively. In addition, the IC50 values of AHP-M-1 and AHP-M-2 were 1.194 mg/mL and 0.67 mg/mL, respectively, which indicated that the DPPH radical scavenging activity of AHP-M-2 was higher than that of AHP-M-1. However, their scavenging activity against DPPH radical was less than that of Vc at the same concentrations. The results demonstrated that AHP-M-1 and AHP-M-2 had good DPPH radical scavenging ability.

#### Hydroxyl radical (•OH) scavenging assay

**Figure 10:**
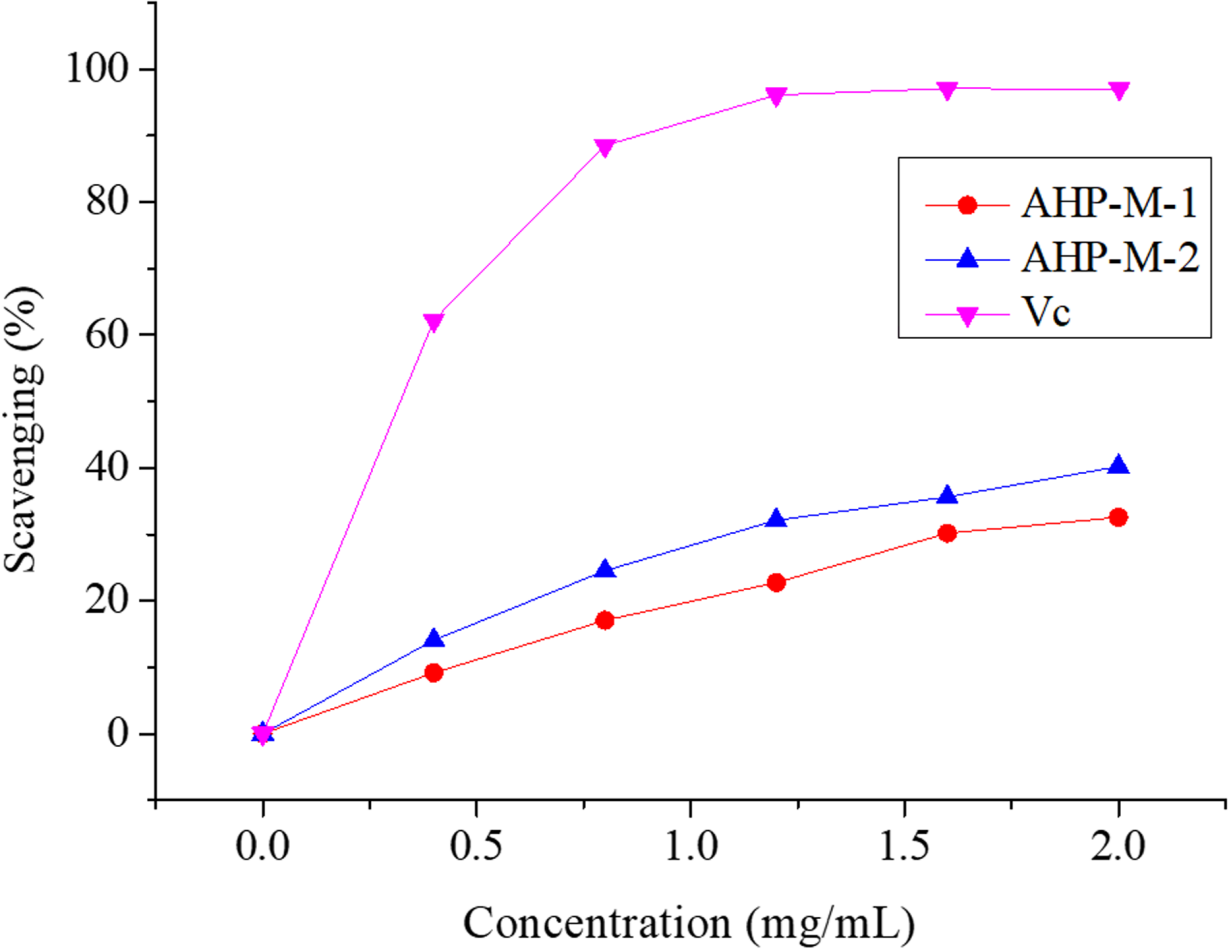
The Hydroxyl radical scavenging ability of purified polysaccharide.

Hydroxyl radicals are very active free radicals that easily react with active substances in the body. Studies have indicated that the excessive accumulation of hydroxyl radicals is associated with cancer risk (Sakanaka, Tachibana & Okada, 2005). The scavenging activities of AHP-M-1, AHP-M-2 and Vc against hydroxyl radicals are shown in Fig. 10. The scavenging activities of AHP-M-1 and AHP-M-2 increased with the concentration of polysaccharides (0–2 mg/mL). However, the scavenging abilities of AHP-M-1 and AHP-M-2 for hydroxyl radicals were significantly lower than that of Vc at the same concentrations in all extracts. At a concentration of 2.0 mg/mL, the scavenging activities were 32.57% and 40.21% for AHP-M-1 and AHP-M-2, respectively, which indicated that the scavenging hydroxyl radical activity of AHP-M-2 was higher than that of AHP-M-1.

#### Superoxide radical (•O^2−^) scavenging assay

**Figure 11:**
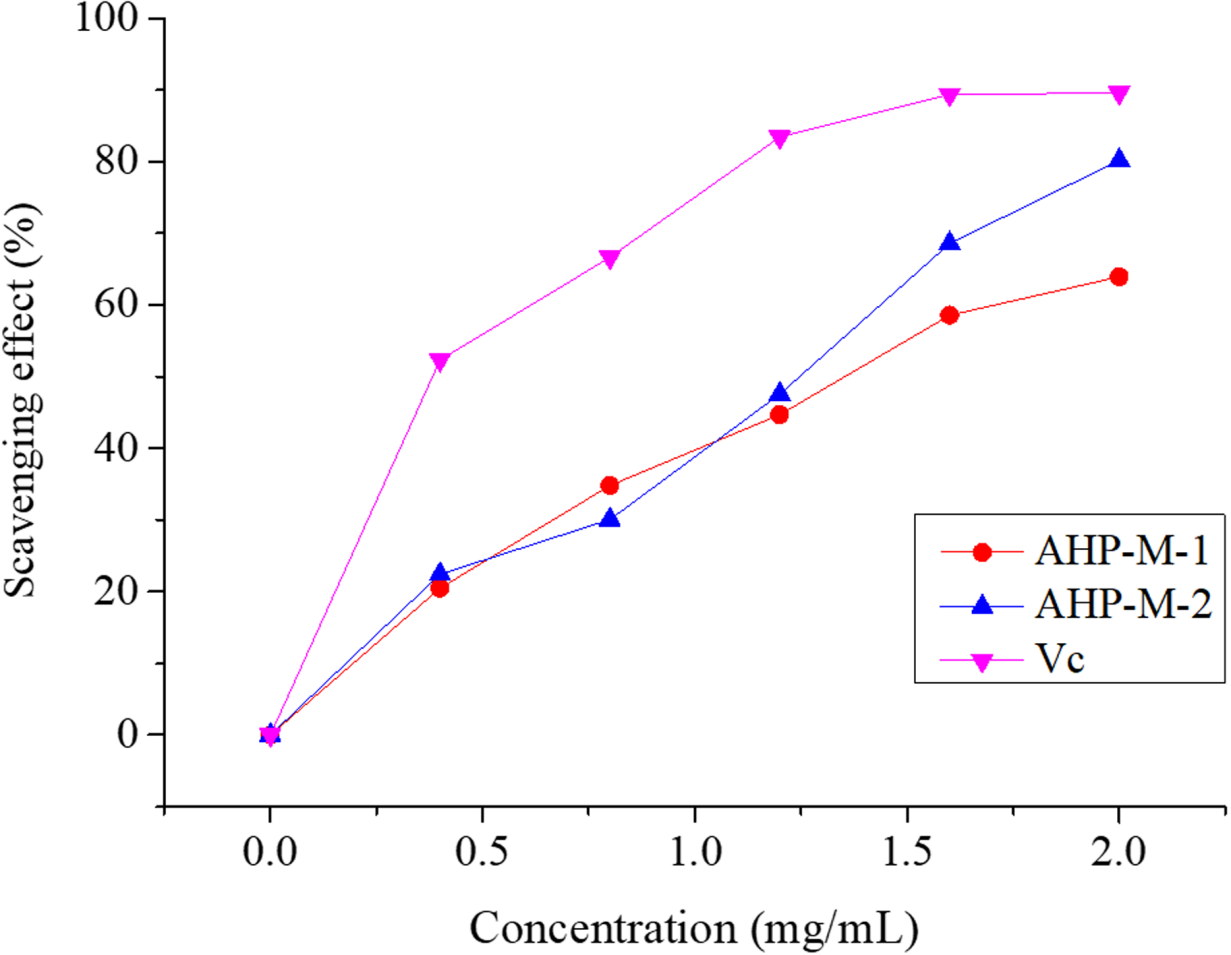
The Superoxide radical scavenging ability of purified polysaccharide.

Superoxide radical is known to be very harmful to cellular components as a precursor of more reactive oxygen species, contributing to tissue damage and various diseases (Ozsoy et al., 2008; Aruoma, Grootveld & Bahorun, 2006). The superoxide radical scavenging abilities of AHP-M-1 and AHP-M-2 are compared with those of the Vc group in Fig. 11. The results showed that both polysaccharides had the potential to scavenge superoxide radicals and that the scavenging rates increased at higher concentrations (0–2 mg/mL). Moreover, at a concentration of 2 mg/mL, the superoxide radical scavenging rates of AHP-M-1 and AHP-M-2 reached 63.95% and 80.21%, respectively.

#### Measurement of the reducing power

**Figure 12:**
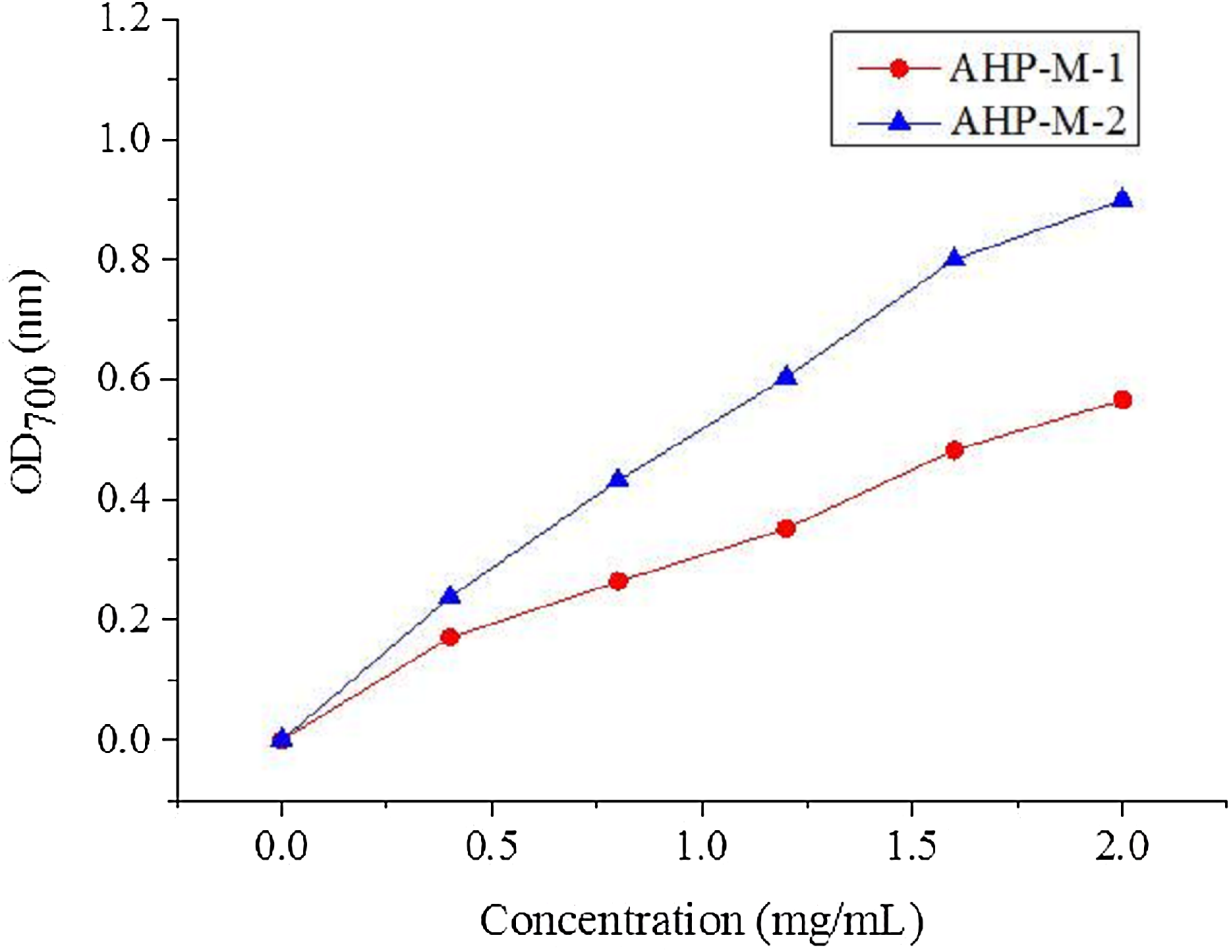
The reducing power of purified polysaccharide.

The reducing power is considered to be an important indicator of antioxidant activity in natural compounds. Antioxidants reduce Fe^3+^ in potassium ferricyanide to Fe^2+^, which can be monitored by the formation of Perl’s Prussian blue at 700 nm. It has also been reported that the deeper the color is, the stronger the reducing power of the substance, and the better the antioxidant activity. The reducing powers of AHP-M-1 and AHP-M-2 are shown in Fig. 12 The results showed that the reducing power increased with concentration. Moreover, at a concentration of 2 mg/mL, the reducing power of AHP-M-1 and AHP-M-2 reached 0.57 and 0.90, respectively.

#### Total antioxidant activity assay

The total antioxidant activity assay is usually performed to determine the antioxidant capacities of natural products such as polyphenols and polysaccharides. The T-AOC in AHP-M-1 and AHP-M-2 was measured according to the T-AOC kit. The results showed that the T-AOC per milligram of AHP-M-1 was 2.2 and the T-AOC per milligram of AHP-M-2 was 6.42. Moreover, the total radical scavenging activity of AHP-M-2 was higher than those of AHP-M-1 and Vc (4.5).

## Discussion

In recent years, polysaccharides have received increasing attention as new sources in the food and pharmaceutical industries. Therefore, the objective of this study was to extract plant polysaccharides from *A. hybridus* L. by a microwave-assisted method and analyze the compounds’ structures and antioxidant activities.

It has been shown that the extraction procedure has a significant impact on the yield and structural characteristics of polysaccharides, as well as their biological activities (Dong et al., 2016). MAE is considered to be an efficient polysaccharide extraction method. Cheng et al. (2015) found that the fruit of *Schisandra chinensis Baill* polysaccharide was extracted by different methods, MAE was more efficient than enzyme-assisted extraction (EAE), Ultrasonic-assisted extraction (UAE) and Heat reflux extraction (HRE). Studies have shown that the extraction efficiency of polysaccharides is related to physical changes in plant tissue in the cell wall (Hemwimon, Pavasant & Shotipruk, 2007). In order to investigate the effect of MAE on the surface structure of crude polysaccharides and to understand the extraction mechanisms, the microstructure of AHP-M was observed by SEM imaging of the product of MAE. The results showed bubble structures or wrinkles on the surface structure of the material, which was related to the microwave processing method. Considerable pressure builds up inside materials during the MAE process (Ying, Han & Li, 2011). This high pressure dramatically alters the physical properties of the cell wall, leading to its destruction, which makes the chemicals in the cell easier to release into the surrounding solvent than other methods (Dong et al., 2016; Cheng et al., 2015). The SEM analysis provided strong evidence that MAE is an effective method for extracting polysaccharides.

It has been found that the generation of reactive oxygen species (ROS) and the corresponding response to oxidative stress are critical factors in the outbreak of several human diseases (Lee & Lee, 2006). Antioxidants have vital functions against ROS in the biological system (Zhang et al., 2015b). Studies concerning the antioxidant and/or scavenger activities of crude plant polysaccharide extracts and purified polysaccharides from various medicinal plants have indicated that these plant carbohydrates show significant antioxidant activities and may be explored as potential antioxidants (Burns et al., 2001; Kovacic & Jacintho, 2001). In the present study, the capacities (DPPH radical scavenging, hydroxyl radical scavenging, superoxide radical scavenging, reducing power and total antioxidant) of AHP-M-1 and AHP-M-2 were studied. The results showed that AHP-M-2 exhibited stronger antioxidant activity than AHP-M-1 against DPPH, superoxide radical and hydroxyl radicals, which could be related to differences in the protein content, monosaccharide composition and chemical structure. Compared to polysaccharides isolated from potato peel, a medicinal plant, AHP-M-2 had higher antioxidant activity (Xu et al., 2019).

Based on the published literature, the antioxidant activity of natural polysaccharides might be related to their composition, molecular weight, water solubility, monosaccharide component, structure of chain conformation, polarity, and intramolecular hydrogen bonds (Raza et al., 2016; Shi et al., 2017). Therefore, the antioxidant activity of polysaccharides is not attributable to a single factor, but to a combination of many factors. Additionally, a previous study reported that the biological activity of polysaccharides was related to their molecular weight (Chen, Wu & Huang, 2013). Liu et al. (2016) found that polysaccharides with a relatively high molecular weight exhibit strong biological activity, which was also consistent with the reported results. However, some researchers have reported that polysaccharides with lower molecular weights present stronger reducing power (Li et al., 2016) It might be concluded that there is no obvious relationship between molecular weight and antioxidant activity. Uronic acids are acknowledged to play an important role in antioxidant activity. Based on previous reports, polysaccharides with a higher content of uronic acids possess stronger antioxidant activities (He et al., 2016). The uronic acid contents of AHP-M-1 and AHP-M-2 were 32.38% and 40.13%, respectively, which was consistent with the results of this experiment. Some studies revealed that mannose and galactose were the key monosaccharide components that resulted in the desired immunological activity (Zhang et al., 2016). The present results indicated that AHP-M-1 and AHP-M-2 were heteropolysaccharides; AHP-M-1 was rich in mannose, whereas AHP-M-2 was rich in galactose and glucose, and these characteristics may be responsible for their immunostimulatory activities. The monosaccharides of polysaccharides extracted from southern and northern *S. chinensis* were mainly composed of galactose and glucose; this composition is similar to that of AHP-M-2 (Yue et al., 2017).

These results indicated that AHP-M can be regarded as a new type of natural antioxidant to be used in functional foods. Although the antioxidant activity found in an in vitro experiment is only indicative of the potential health benefit, these results remain important as the first step in screening the antioxidant activity of *A. hybridus* L. Further scientific work in our laboratory is in progress to ensure that the medicinal properties of the plant in *vivo* correlate with its antioxidant activity.

## Conclusions

In the present study, we isolated polysaccharides from *A. hybridus* L. (AHP-M) using MAE. There were obvious differences in the monosaccharide contents of each sample according to GC-MS. The molecular weights of AHP-M-1 and AHP-M-2 were 77.625 kDa and 93.325 kDa, respectively. AHP-M-2 had higher antioxidant activity in vitro than AHP-M-1. These results demonstrated that AHP-M-1 and AHP-M-2 are potential natural antioxidants for use in functional foods and thus could be valuable with regard to the utilization of *A. hybridus* L. for the extraction of active polysaccharides. However, the structure-activity relationship and the mechanism of antioxidant activities for AHP-M remain to be explored in future studies.

## Supplemental Information

10.7717/peerj.9077/supp-1 Supplemental Information 1 The original GC-MS data of AHP-M-1 and AHP-M-2.

10.7717/peerj.9077/supp-2 Supplemental Information 2 Raw data.

## Additional Information and Declarations

### Data Availability

The following information was supplied regarding data availability: The raw measurements are available in the Supplemental Files.
