# Supplementary material for: Purification, characterization and antioxidant activities in vitro of polysaccharides from Amaranthus hybridus L
Source: PeerJ. 2020 Apr 29;8:e9077. doi: 10.7717/peerj.9077 (PMC7195838; doi:10.7717/peerj.9077)

Due to laboratory equipment limitations, the input chromatogram was in Chinese format, which we translated. The following is the translated version:

Methods

annotation

annotation

[GC-2010]

Oven temperature: 40 ℃

Injection temperature: 290.00 ℃

Injection mode: shunt

Flow control mode: pressure

Pressure: 49.5 kPa

Total flow rate: 14.0 mL/min

Column flow rate: 1.00 mL/min

Linear velocity: 36.1 cm/ sec

Purge flow: 3.0 mL/min

Split ratio: 10.0

High pressure injection mode: off

Carrier gas saver: off

Shunt damping fixed: off

Column oven temperature program:

Velocity	temperature (℃)	time (min)
-	40.0	8.00
10.00	100.0	5.00
5.00	220.0	5.00
10.00	280.0	5.00
5.00	300.0	10.00

< The heating unit has been checked >

Column thermostat: Yes

SPL1: yes

MS: yes

<Detector (FTD) check completed >

< Baseline move check completed >

<Injection flow check completed>

SPL1 carrier gas: yes

SPL1 purge: yes

< APC flow check is completed >

<Detector APC flow check completed >

External waiting: No

Equilibrium time: 3.0 min

[GC program]

[GCMS-QP2010 Plus]

Ion source temperature: 200.00 ℃

Interface temperature: 220.00 ℃

Solvent delay time: 3.80 min

Detector gain mode: relative

Detector gain: 0.00 kV

Threshold: 0

[table] MS

-- group 1- event 1--

Start time: 4.00 min

End time: 73.00 min

Mode of ACQ: Scan

Interval: 0.50 sec

Scanning speed: 1428

Start m/z: 33.00

End m/z: 700.00

Sample injection unit: GC

[MS]

Use MS program: off

Chromatographic figure 4 D:\dxd2016\2017。01\(16)W0831B-0.3w.qgd

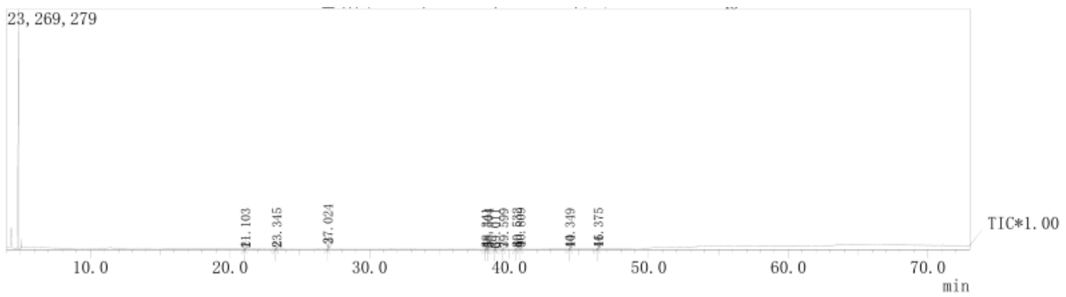

Chromatographic figure 4 D:\dxd2016\2017。01\(16)W0831B-0.3w.qgd

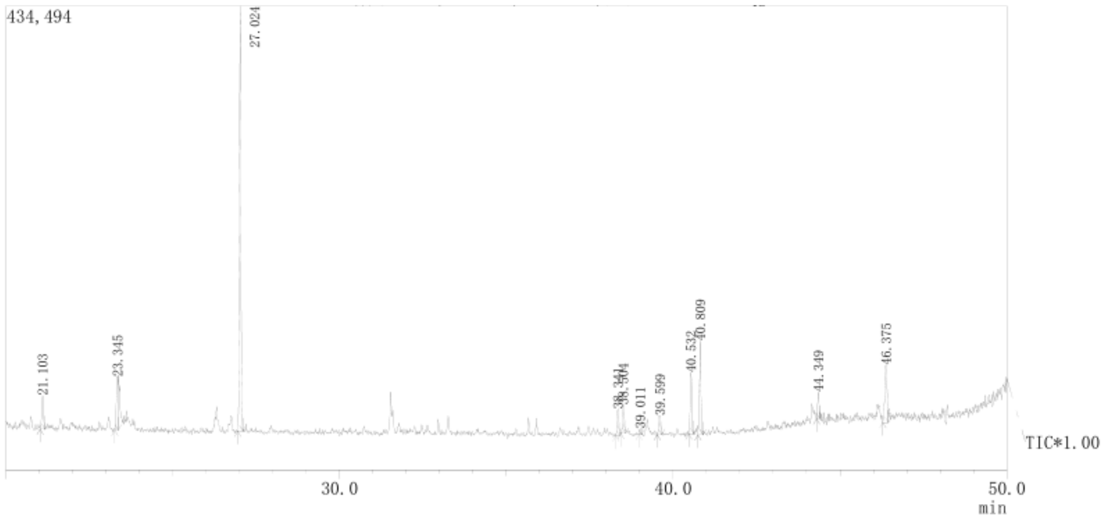

Peak table TIC

Peak number	retains time	I.Time	F.Time	peak area	peak area %	peak height	peak height %	product/hei-ght	mark	name
1	21.103	21.025	21.167	114120	4.54	31372	4.02	3.63	MI	
2	23.345	23.267	23.367	186051	7.40	50220	6.44	3.70	MI	
3	27.024	26.950	27.100	1166794	46.41	398250	51.09	2.92	MI	
4	38.341	38.283	38.392	74435	2.96	24901	3.19	2.98	MI	
5	38.504	38.433	38.575	98066	3.90	26852	3.45	3.65	MI	
6	39.011	38.975	39.050	12183	0.48	5540	0.71	2.19	MI	
7	39.599	39.525	39.658	67947	2.70	17821	2.29	3.81	MI	
8	40.532	40.467	40.617	186669	7.42	57397	7.36	3.25	MI	
9	40.809	40.742	40.892	251237	9.99	88179	11.31	2.84	MI	

10	44.349	44.300	44.392	75299	2.99	24800	3.19	3.02	MI
11	46.375	46.285	46.450	281452	11.19	54028	6.93	5.20	MI
				2514253	100.00	779440	100.00		

Spectral library

<< target component >>

Line number: 1 retention time: 21.100(number of scans: 2053) quality peak: 353 base peak: 43.05(8075)

Original pattern: Averaged 21.025-21.192 (2044-2064) background model: 21.017 (2043).

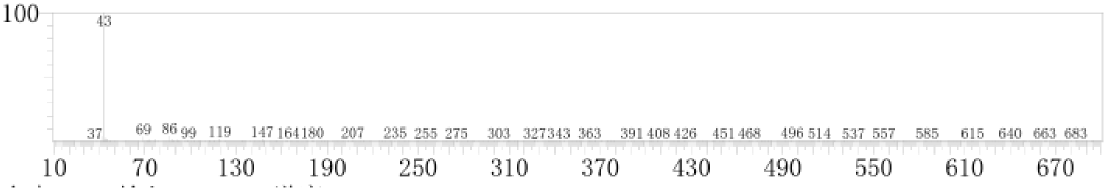

Hit #: 1 input: 43631 spectrum library: NIST08.LIB

SI: 87 molecular formula: $C_9H_{14}O_5$ CAS: 73972-39-1 mol mass: 202 retention index: 1258

Component name: Propanedioic acid, oxo-, bis(1-methylethyl) ester $$Diisopropyl 2-oxomalonat

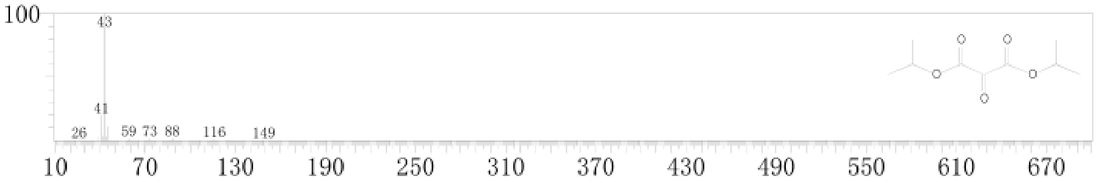

Hit #: 2 input: 803 spectrum library: NIST08.LIB

SI: 86 molecular formula: $C_4H_6O_2$ CAS: 108-05-4 mol mass: 86 retention index: 576

Acetic acid ethenyl ester $$Acetic acid vinyl ester $$Viny

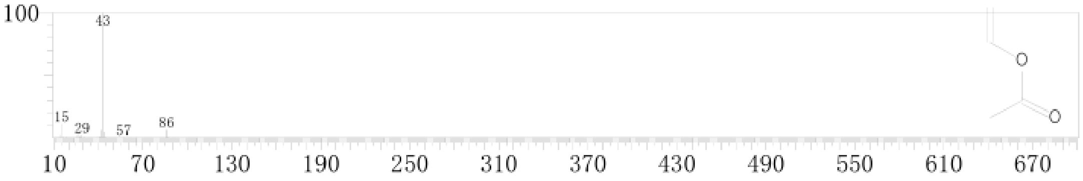

Hit #: 3 input: 4476 spectrum library: NIST08.LIB

SI: 86 molecular formula: $C_5H_8O_3$ CAS: 592-20-1 mol mass: 116 retention index: 822

Component name: 2-propanone, 1-(acetyloxy)- $$2-propanone, 1-hydroxy-, acetate $$Acetol ace

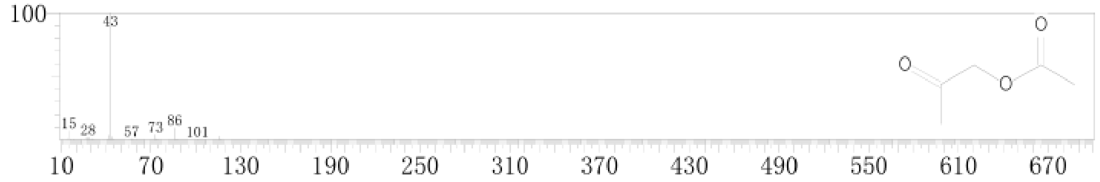

<< target component >>

Line number: 2 retention time: 23.342(number of scans: 2322) quality peak: 362 base peak: 43.05(17039)

Original pattern: Averaged 23.250 23.367 (2311-2325) background model: 23.267 (2313).

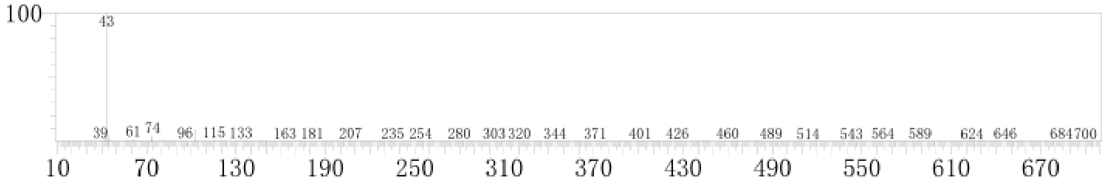

Hit #: 1 input: 8808 spectrum library: NIST08.LIB

SI: 89 molecular formula: $C_5H_{10}O_4$ CAS: 106-61-6 mol mass: 134 retention index: 1091

Components: 1, 2, 3-propanetriol, 1-acetate $$Acetin, 1-mono-$$.alpha. -monoacetin $$Gly

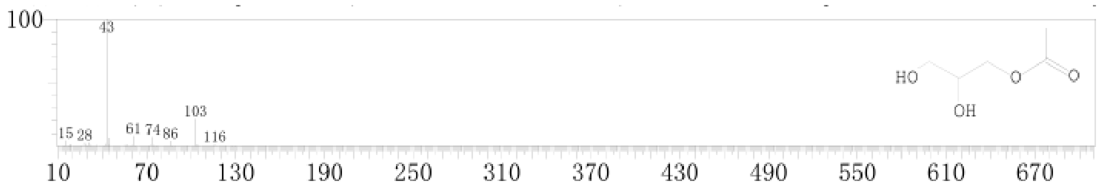

Hit #:2 input: 44765 spectrum library: NIST08.LIB

SI: 88 molecular formula: $C_8H_{12}O_6$ CAS: 2983-35-9 mol mass: 204 retention index: 1254

Component name: 1, 1, 2-triacetoxyethane $$1, 1, 2-ethanetriol, triacetate $$1, 2-bis (acetyloxy)

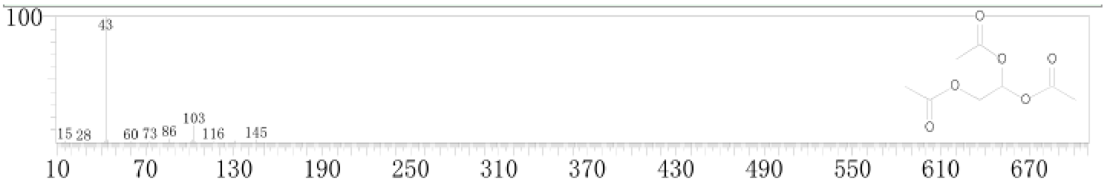

Hit #: 3 input: 4470 library: NIST08.LIB

SI: 88 molecular formula: $C_5H_8O_3$ CAS: 6387-89-9 mol mass: 116 retention index: 776

Component name: 1, 2-propyl acetate $$Oxiranemethanol, acetate $$Acetic acid, oxiran

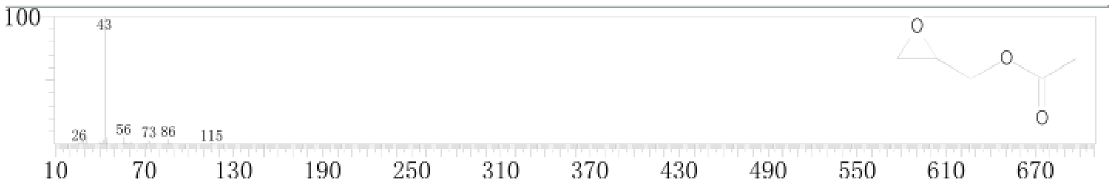

<< target component >>

Line number: 3 retention time: 27.025(number of scans: 2764) quality peak: 377 base peak: 43.05(105178)

Original pattern: Averaged 26.958 27.083 (2756-2771) background model: 26.942 (2754).

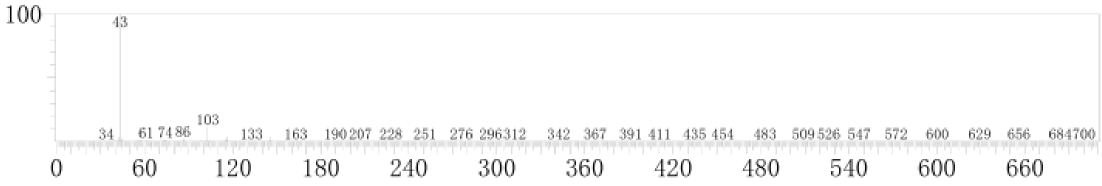

Hit #: 1 input: 54008 spectrum library: NIST08.LIB

SI: 96 molecular formula: $C_9H_{14}O_6$ CAS: 102-76-1 mol mass: 218 retention index: 1354

Component name: Triacetin $$1, 2, 3-propanetriol, triacetate $$Acetin, tri- $$Enzactin $$Fu

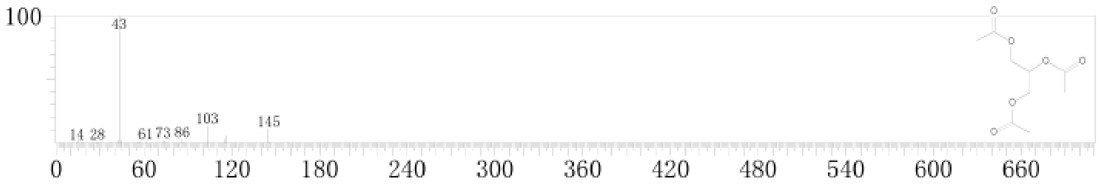

Hit #: 2 input: 44765 spectrum library: NIST08.LIB

SI: 94 molecular formula: $C_8H_{12}O_6$ CAS: 2983-35-9 mol mass: 204 retention index: 1254

Component name: 1, 1, 2-triacetoxyethane $$1, 1, 2-ethanetriol, triacetate $$1, 2-bis (acetyloxy)

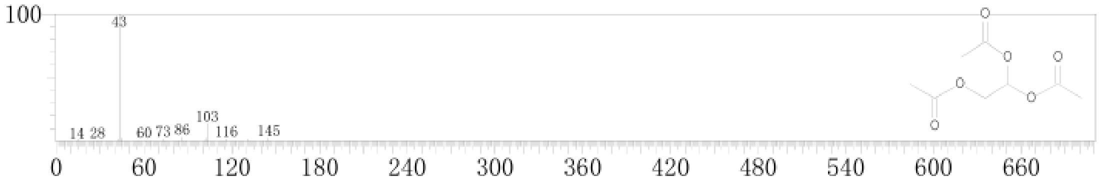

Hit #:3 input: 28369 spectrum library: NIST08.LIB

SI: 93 molecular formula: $C_7H_{12}O_5$ CAS: 25395-31-7 mol mass: 176 retention index: 1230

Components: 1, 2, 3-propanetriol, diacetate $$Acetin, di-$$Diacetin $$Diacetylglycerol

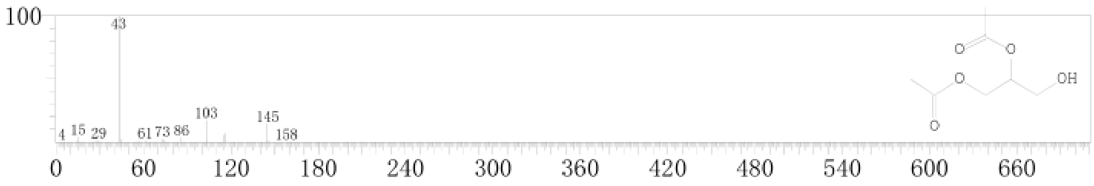

<< target component >>

Line number: 4 retention time: 38.342(scan number: 4122) quality peak: 366 base peak: 43.05(4959)

Original pattern: Averaged 38.292 38.400 (4116-4129) background model: 38.283 (4115).

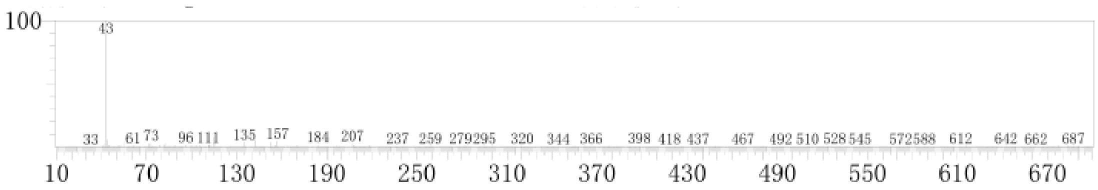

Hit #: 1 input: 133652 spectrum library: NIST08.LIB

SI: 83 molecular formula: $C_{14}H_{20}O_9$ CAS: 27821-11-0 mol mass: 332 retention index: 2081

Component name: alpha.-l-mannopyranose, 6-deoxy-, tetraacetate $$1, 2, 3, 4-tetra -o-acetyl-6-d

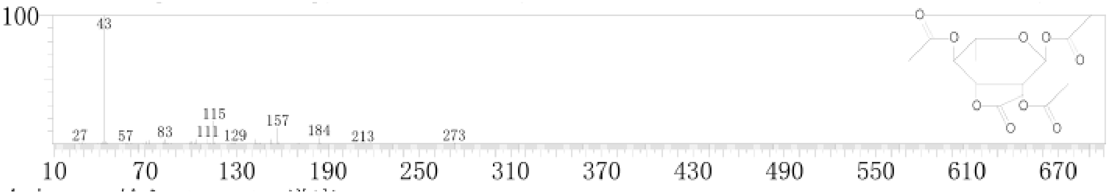

Hit #: 2 input: 133651 spectrum library: NIST08.LIB

SI: 79 molecular formula: $C_{14}H_{20}O_9$ CAS: 0-00-0 molar mass: 332 retention index: 2081

Tetraacetyl. Beta.-d-rhamnose

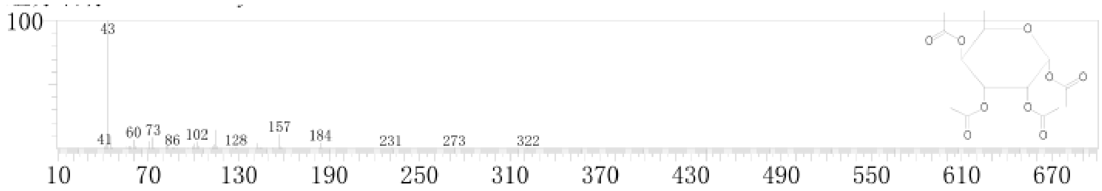

Hit #: 3 input: 122324 spectrum library: NIST08.LIB

SI: 77 molecular formula: $C_{13}H_{17}NO_8$ CAS: 34360-56-0 molar mass: 315 retention index: 2036

Component name: lyxonitrile, 2, 3, 4, 5-tetraacetate, d-

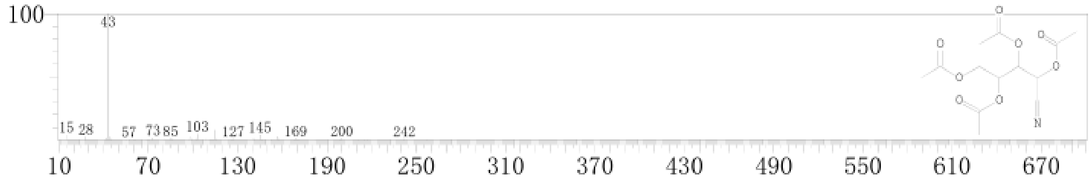

<< target component >>

Line number: 5 retention time: 38.508(number of scans: 4142) quality peak: 379 base peak: 43.00(7969)

Original pattern: Averaged 38.433 38.542 (4133-4146) background model: 38.433 (4133).

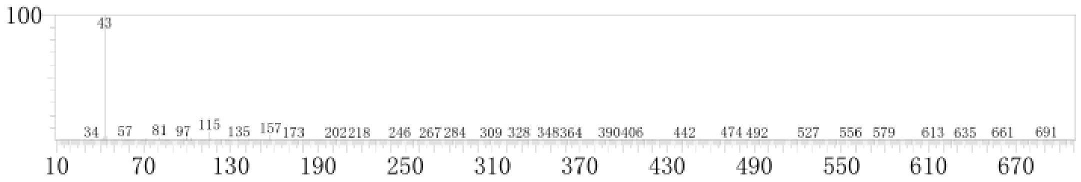

Hit #: 1 input: 82982 library: NIST08.LIB

SI: 85 molecular formula: $C_{11}H_{16}O_7$ CAS: 19200-32-9 mol mass: 260 retention index: 1692

Component name: Xylitol, 1, 5-anhydro-, triacetate $$2,3, 4-trii-o-acetyl-1,5-anhydropentitol #

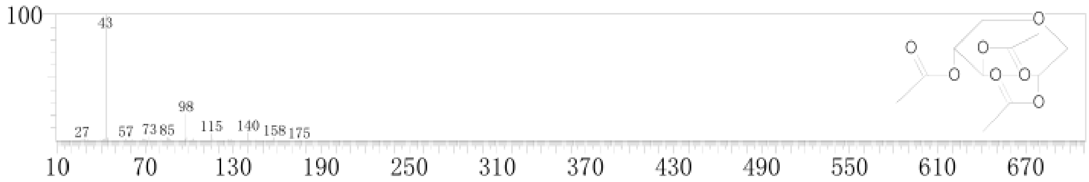

Hit #: 2 input: 122324 spectrum library: NIST08.LIB

SI: 84 molecular formula: $C_{13}H_{17}NO_8$ CAS: 34360-56-0 molar mass: 315 retention index: 2036

Component name: lyxonitrile, 2, 3, 4, 5-tetraacetate, d-

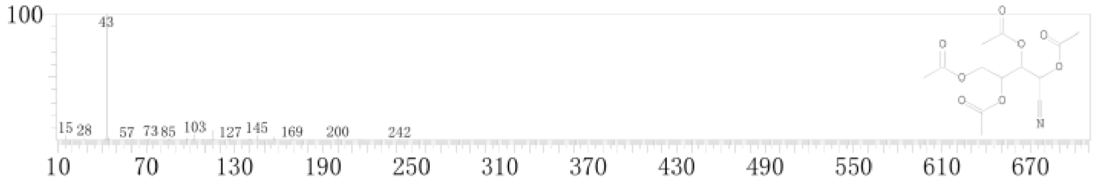

Hit #:3 input: 18946 library: NIST08.LIB

SI: 84 molecular formula: $C_7H_{10}O_4$ CAS: 869-29-4 mol mass: 158 retention index: 978

Component name: 2-propene-1, 1-diol, diacetate $$Acrolein, diacetate $$Allylidene acetate $$$

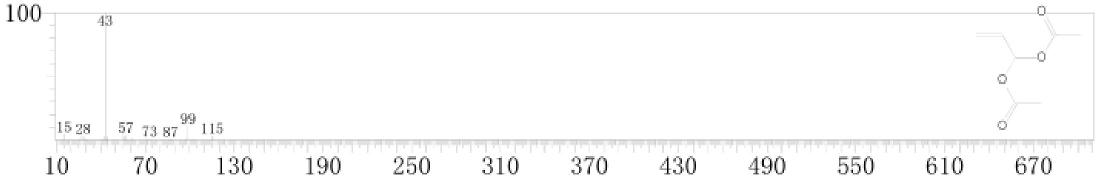

<< target component >>

Line number: 6 retention time: 39.008(number of scans: 4202)

Original pattern: Averaged 38.983 39.033 (4199-4205) background model: 38.967 (4197).

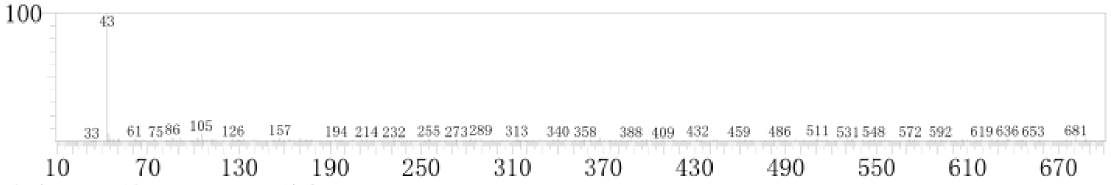

Hit #: 1 input: 35297 spectrum library: NIST08.LIB

SI: 69 molecular formula: $C_9H_{16}O_4$ CAS: 7371-86-0 mol mass: 188 retention index: 1122

Component name: 2, 4-diacetoxypentane $$3-(acetyloxy-1-methylbutyacetate # $$$

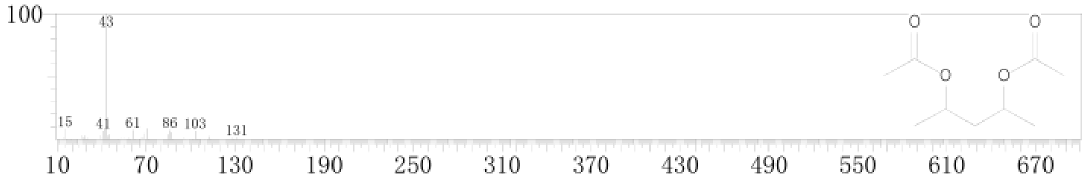

Hit #: 2 input: 143549 spectrum library: NIST08.LIB

SI: 69 molecular formula: $C_{14}H_{20}O_{10}$ CAS: 13240-99-8 mol mass: 348 retention index: 2110

Component name: Methyl (Methyl -2, 3, 4-tri-o-acetyl. Beta. D-galactofuranosid) urinate

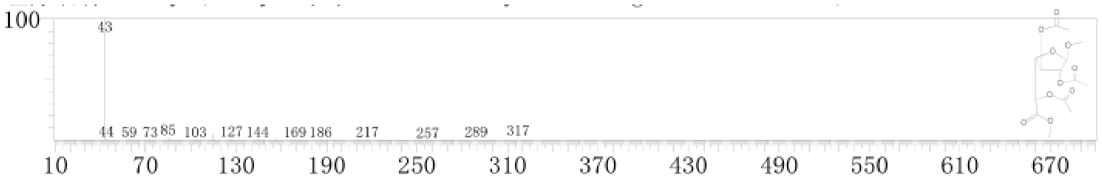

Hit #: 3 input: 7778 spectrum library: NIST08.LIB

SI: 69 molecular formula: $C_6H_{10}O_3$ CAS: 0-00-0 mol mass: 130 retention index: 857

Component name: 3-hydroxy-2-butanone, acetate

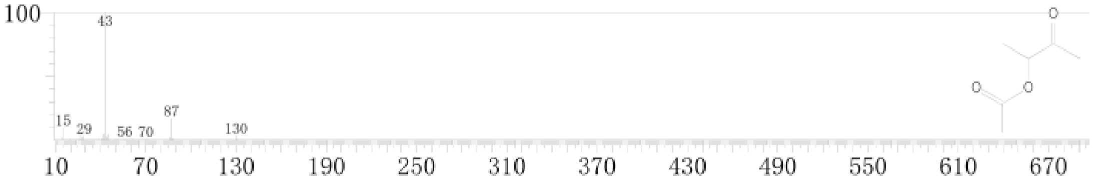

<< target component >>

Line number: 7 retention time: 39.600(number of scans: 4273) quality peak: 355 base peak: 43.05(3876)

Original pattern: Averaged 39.550 39.633 (4267-4277) background model: 39.542 (4266).

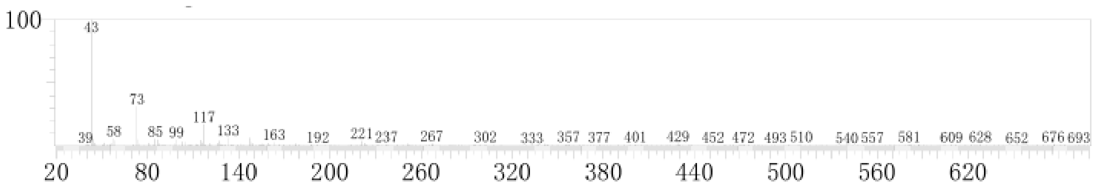

Hit #:1 input: 133053 spectrum library: NIST08.LIB

SI: 70 molecular formula: $C_{14}H_{21}NO_8$ CAS: 59061-07-3 mol mass: 331 retention index: 2055

Component name: 4, 5, 6-tri-o-acetyl-2, 3-di-o-methyl -d-mannonitrile

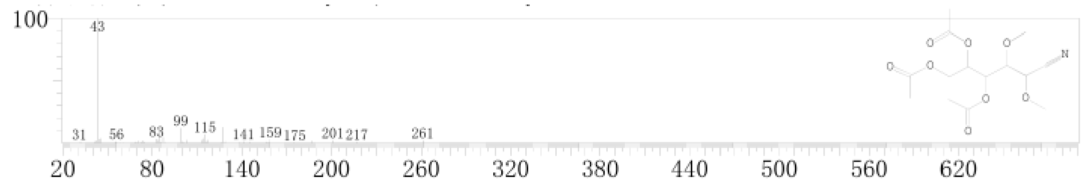

Hit #: 2 input: 29401 library: NIST08.LIB

SI: 70 molecular formula: $C_7H_{14}O_5$ CAS: 36680-67-8 mol mass: 178 retention index: 1194

Component name: Acetic acid, 3-hydroxy-2, 2-dimethoxy-propyl ester $$3-hydroxy-2, 2-dimethoxyp

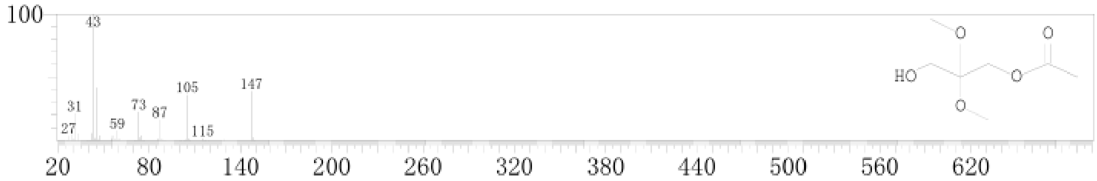

<< target component >>

Line number: 7 retention time: 39.600(number of scans: 4273) quality peak: 355 base peak: 43.05(3876)

Original pattern: Averaged 39.550 39.633 (4267-4277) background model: 39.542 (4266)

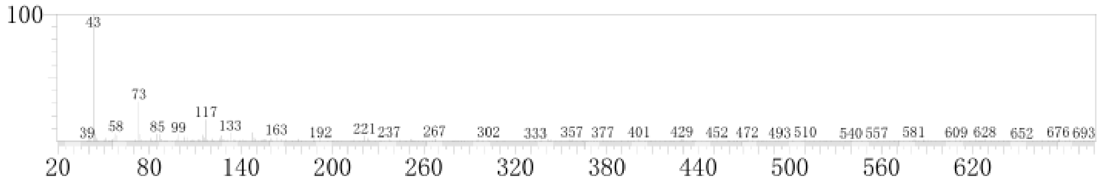

Hit #: 3 input: 44874 library: NIST08.LIB

Molecular formula: $C_9H_{20}O_3Si$ CAS: 108536-14-7 mol mass: 204 retention index: 1011

Component name: 1, 2-dioxetane, 3, 4, 4-trimethyl-3-[[(trimethylsilyl)oxy]methyl]- $$(3,4, 4-tri

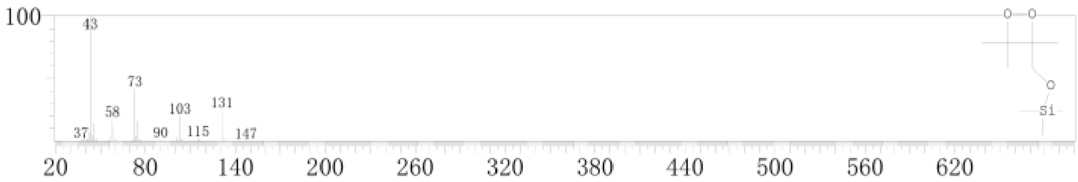

<< target component >>

Line number: 8 retention time: 40.533(scan number: 4385) mass peak: 380 base peak: 43.05(11502)

Original pattern: Averaged 40.483 40.600 (4379-4393) background model: 40.467 (4377).

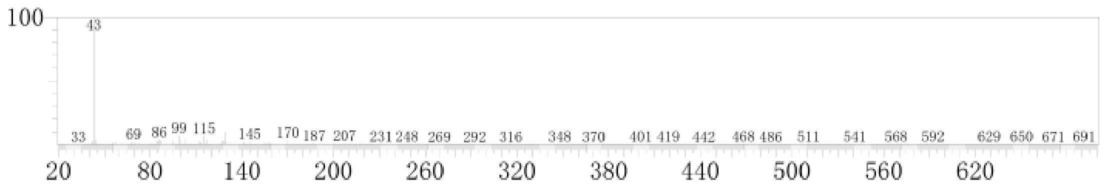

Hit #: 1 input: 158315 spectrum library: NIST08.LIB

SI: 87 molecular formula: $C_{16}H_{24}O_{10}$ CAS: 15086-09-6 mol mass: 376 retention index: 2256

Component name: 2-deoxysorbitol pentaacetate $$1, 2, 3, 4, 6-penta-o-acetyl-5-deoxyhexitol # $$

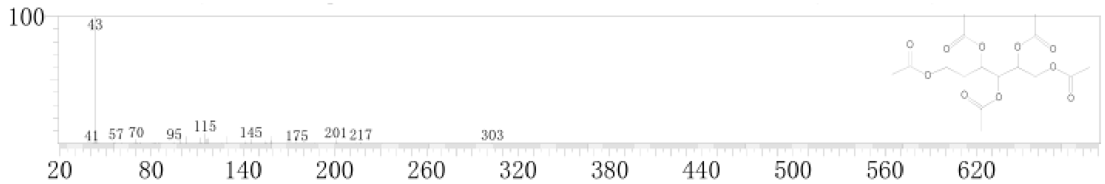

Hit #: 2 input: 158316 library: NIST08.LIB

SI: 86 molecular formula: $C_{16}H_{24}O_{10}$ CAS: 49560-35-2 mol mass: 376 retention index: 2192

Component name: d-arabino-hexitol, 5-deoxy-, pentaacetate $$1, 2, 3, 4, 5-penta-o-acetyl-6-deoxy

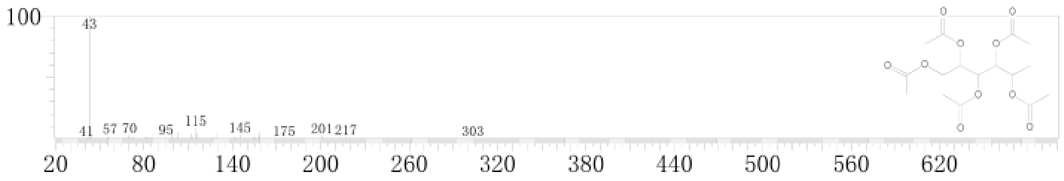

Hit #:3 input: 104455 library: NIST08.LIB

SI: 85 molecular formula: $C_{12}H_{18}O_8$ CAS: 7208-40-4 mol mass: 290 retention index: 1755

Component name: 1, 2, 3, 4-butanetetrol, tetraacetate, (R*, S*)- $$2, 3-bis (acetyloxy)-1-[(acety)

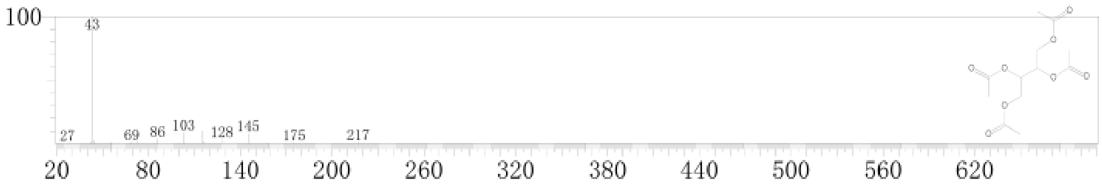

<< target component >>

Line number: 9 retention time: 40.808(number of scans: 4418) quality peak: 377 base peak: 43.05(13025)

Original pattern: Averaged 40.750 40.883 (4411-4427) background model: 40.750 (4411).

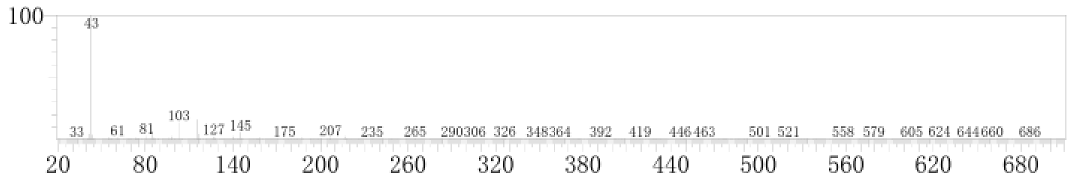

Hit #: 1 input: 104458 spectrum library: NIST08.LIB

SI: 89 molecular formula: $C_{12}H_{18}O_8$ CAS: 73977-54-5 molar mass: 290 retention index: 1755

Component name: 1, 2, 3, 4-butanetetrol, tetraacetate $2, 3-bis (acetyloxy)-1-[(acetyloxy)methy

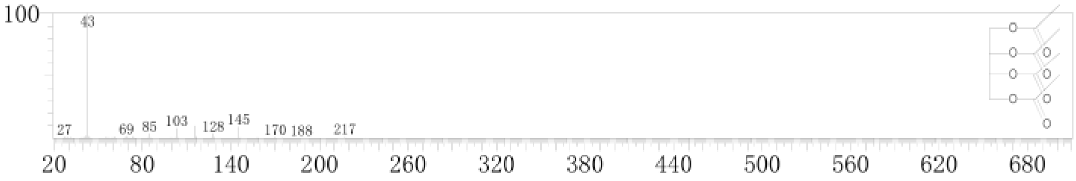

Hit #: 2 input: 104456 spectrum library: NIST08.LIB

SI: 89 molecular formula: $C_{12}H_{18}O_8$ CAS: 49560-29-4 molar mass: 290 retention index: 1755

Component name: 1, 2, 3, 4 - Butanetetrol tetraacetate, [s - (R * R *)] - $$2, 3 - Bis (acetyloxy) - 1 - [(ac

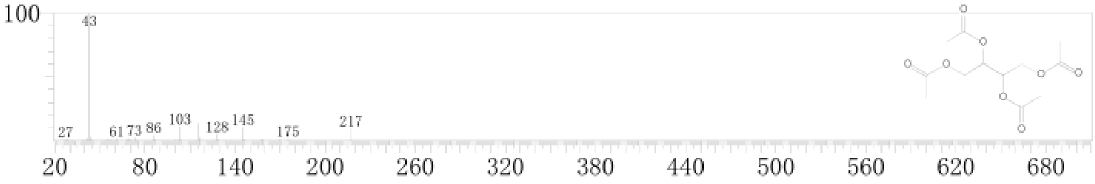

Hit #: 3 input: 104455 library: NIST08.LIB

SI: 88 molecular formula: $C_{12}H_{18}O_8$ CAS: 7208-40-4 mol mass: 290 retention index: 1755

Component name: 1, 2, 3, 4-butanetetrol, tetraacetate, (R*, S*)- $$2, 3-bis (acetyloxy)-1-[(acetyl)

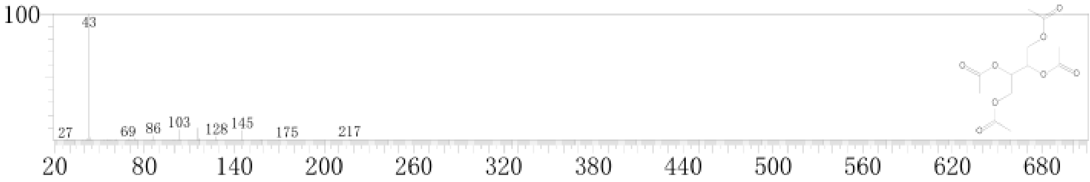

<< target component >>

Line number: 10 retention time: 44.350(number of scans: 4843) quality peak: 373 base peak: 43.05(3691)

Original pattern: Averaged 44.283 44.400 (4835-4849) background model: 44.292 (4836).

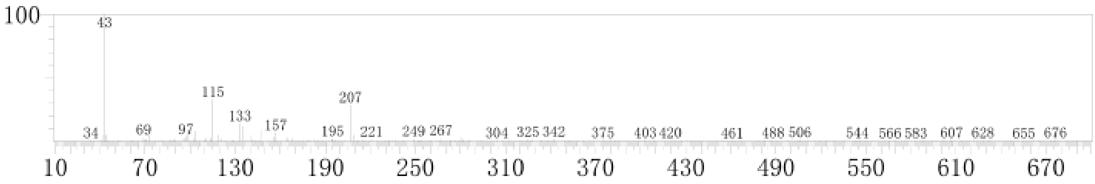

Hit #: 1 input: 164048 spectrum library: NIST08.LIB

SI: 67 molecular formula: $C_{16}H_{22}O_{11}$ CAS: 3891-59-6 mol mass: 390 retention index: 2380

Component name: d-glucose, 2, 3, 4, 5, 6-pentaacetate $$d-glucose pentaacetate $$Glucose pentaa

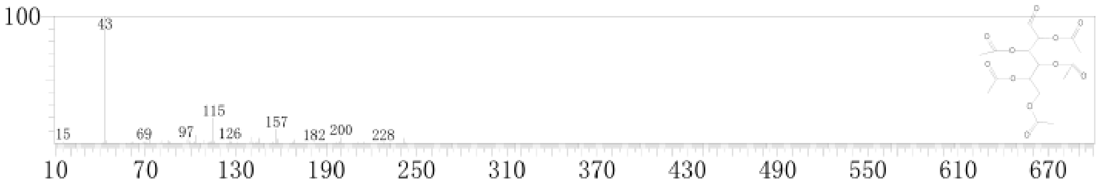

Hit #: 2 input: 164046 spectrum library: NIST08.LIB

SI: 66 molecular formula: $C_{16}H_{22}O_{11}$ CAS: 604-68-2 mol mass: 390 retention index: 2447

Glucopyranose, pentaacetate, alpha. -d-glucopyranose, pentaacetate $$Glucopyranose, pentaacetate, alpha.

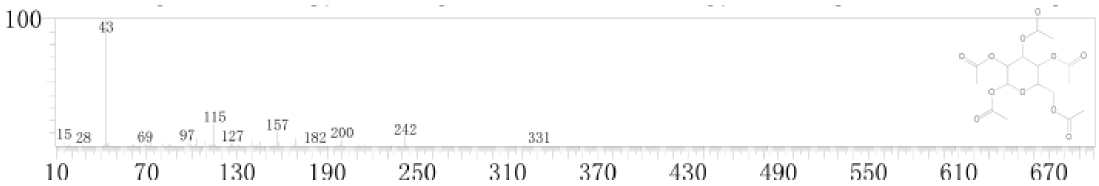

Hit #: 3 input: 143552 library: NIST08.LIB

SI: 66 molecular formula: $C_{14}H_{20}O_{10}$ CAS: 0-00-0 mol mass: 348 retention index: 2307

Component name: Tetra-O-acetyl-d-mannopyranose

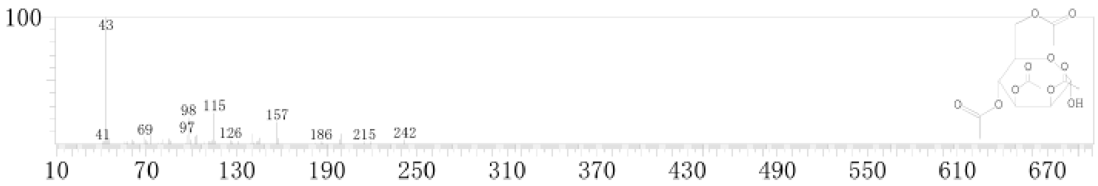

<< target component >>

Line number: 11 retention time: 46.375(scan number: 5086) quality peak: 397 base peak: 43.05(9112)

Original pattern: Averaged 46.283 46.417 (5075-5091) background model: 46.283 (5075).

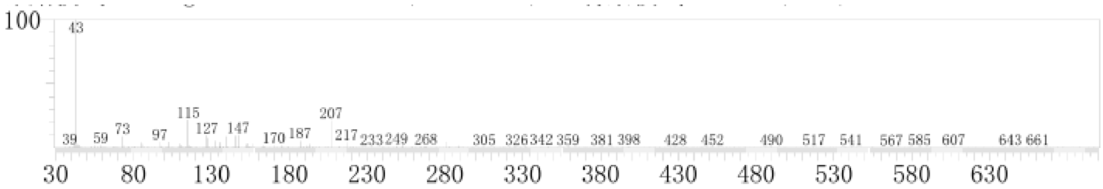

Hit #: 1 input: 176096 library: NIST08.LIB

SI: 79 molecular formula: $C_{18}H_{26}O_{12}$ CAS: 5346-77-0 molar mass: 434 retention index: 2558

Component name: Galactitol, hexaacetate $$1, 2, 3, 4, 5, 6-hexa-o-acetylhexitol # $$

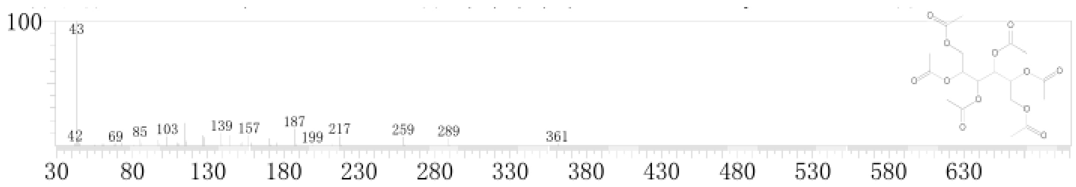

Hit #: 2 input: 176097 spectrum library: NIST08.LIB

SI: 79 molecular formula: $C_{18}H_{26}O_{12}$ CAS: 642-00-2 mol mass: 434 retention index: 2558

Component name: d-mannitol, hexaacetate $$Mannitol, hexaacetate, d-$$Mannitol, hexaacetate

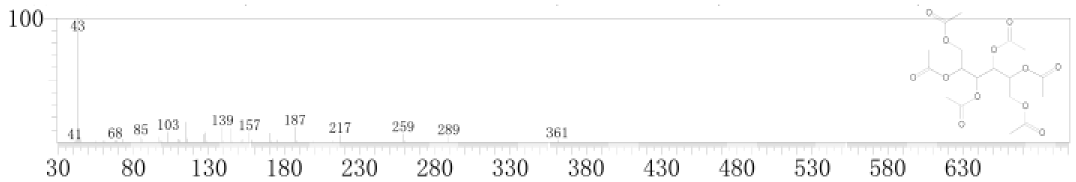

Hit #: 3 input: 176093 spectrum library: NIST08.LIB

SI: 79 molecular formula: $C_{18}H_{26}O_{12}$ CAS: 13443-46-4 mol mass: 434 retention index: 2558

Component name: l-iditol, hexaacetate $$1, 2, 3, 4, 5, 6-hexa-o-acetylhexitol # $$

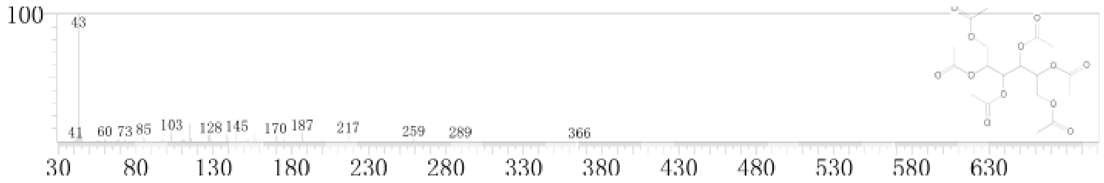

Supplement: Supplemental Information 1 [file peerj-08-9077-s001.zip › original GC-MS data/Translation version---AHP-M-1.pdf]
