# Supplementary material for: Purification, characterization and antioxidant activities in vitro of polysaccharides from Amaranthus hybridus L
Source: PeerJ. 2020 Apr 29;8:e9077. doi: 10.7717/peerj.9077 (PMC7195838; doi:10.7717/peerj.9077)

Due to laboratory equipment limitations, the input chromatogram was in Chinese format, which we translated. The following is the translated version:

Methods

annotation

[GC-2010]

Oven temperature: 40 ℃

Injection temperature: 290.00 ℃

Injection mode: shunt

Flow control mode: pressure

Pressure: 49.5 kPa

Total flow rate: 14.0 mL/min

Column flow rate: 1.00 mL/min

Linear velocity: 36.1 cm/ sec

Purge flow: 3.0 mL/min

Split ratio: 10.0

High pressure injection mode: off

Carrier gas saver: off

Shunt damping fixed: off

Column oven temperature program:

Velocity	temperature (℃)	time (min)
-	40.0	8.00
10.00	100.0	5.00
5.00	220.0	5.00
10.00	280.0	5.00
5.00	300.0	10.00

< The heating unit has been checked >

Column thermostat: Yes

SPL1: yes

MS: yes

<Detector (FTD) check completed >

< Baseline move check completed >

<Injection flow check completed>

SPL1 carrier gas: yes

SPL1 purge: yes

< APC flow check is completed >

<Detector APC flow check completed >

External waiting: No

Equilibrium time: 3.0 min

[GC program]

[GCMS-QP2010 Plus]

Ion source temperature: 200.00 ℃

Interface temperature: 220.00 ℃

Solvent delay time: 3.80 min

Detector gain mode: relative

Detector gain: 0.00 kV

Threshold: 0

[table] MS

-- group 1- event 1--

Start time: 4.00 min

End time: 73.00 min

Mode of ACQ: Scan

Interval: 0.50 sec

Scanning speed: 1428

Start m/z: 33.00

End m/z: 700.00

Sample injection unit: GC

[MS]

Use MS program: off

Chromatographic figure 6 D:\dxd2016\2017。01\ (16)W0831B-0.8w.qgd

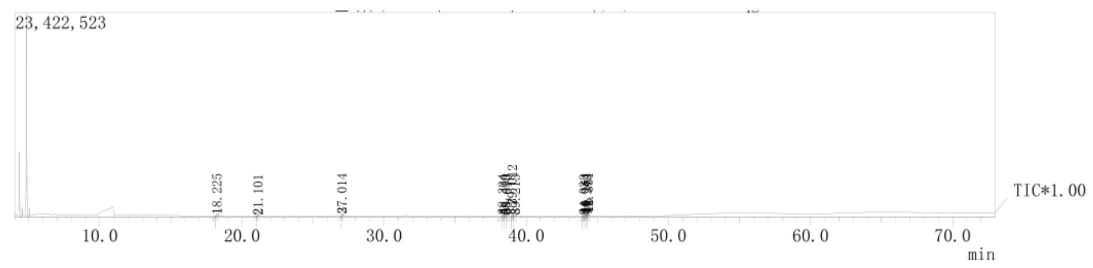

Chromatographic figure 6 D:\dxd2016\2017。01\ (16) W0831B-0.8w.qgd

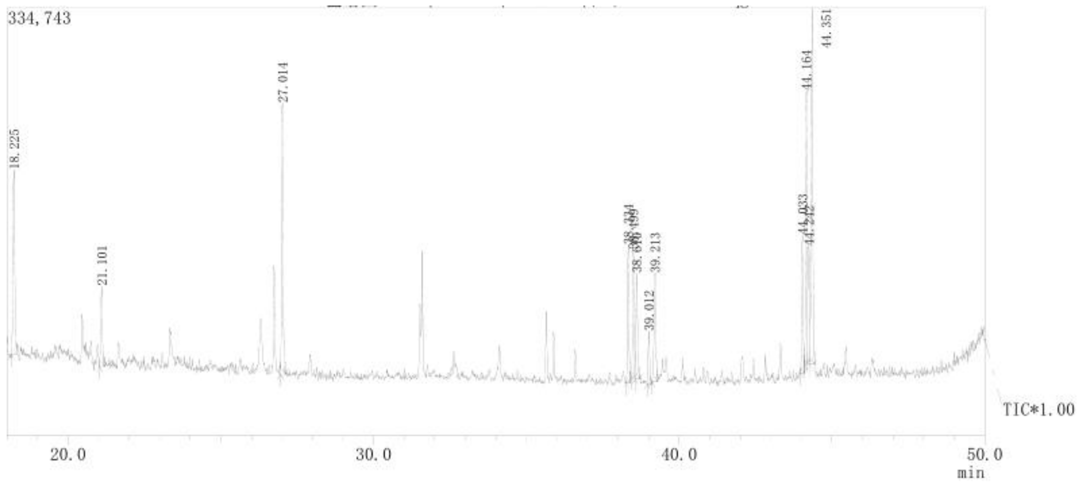

Peak table TIC

Peak number	retains time	I.Time	F.Time	peak area	peak area %	peak height	peak height %	product/height	mark	name
1	18.225	18.142	18.317	616377	11.43	144367	9.45	4.26	MI	
2	21.101	21.025	21.175	227698	4.22	62999	4.12	3.61	MI	
3	27.014	26.958	27.108	714264	13.25	211925	13.86	3.37	MI	
4	38.334	38.267	38.392	319731	5.93	108282	7.08	2.95	MI	
5	38.499	38.433	38.550	355008	6.58	99906	6.54	3.55	MI	
6	38.610	38.558	38.708	302756	5.62	82878	5.42	3.65	MI	
7	39.012	38.950	39.067	136037	2.52	41578	2.72	3.27	MI	
8	39.213	39.117	39.292	353318	6.55	85176	5.57	4.14	MI	
9	44.033	43.967	40.083	349734	6.49	108549	7.10	3.22	MI	
10	44.164	44.100	44.208	756218	14.03	214402	14.03	3.52	MI	

11	44.242	44.208	44.283	301884	5.60	89747	5.87	3.36	MI
12	44.351	44.283	44.433	958449	17.788	278689	18.23	3.43	MI
				5391474	100.00	1528498	100.00		

Spectral library

<< target component >>

Line number: 1 retention time: 18.225(number of scans: 1708) quality peak: 347 base peak: 43.00(14100)

Original pattern: Averaged 18.108 18.317 (1694-1719) background model: 18.142 (1698).

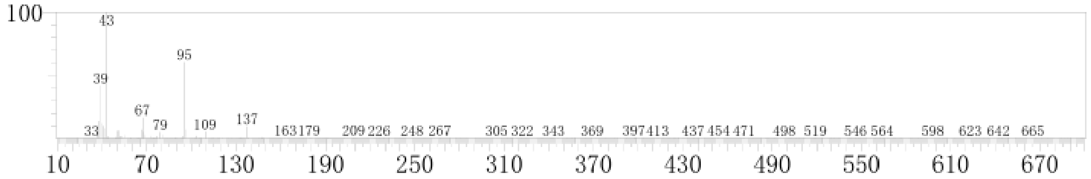

Hit #: 1 input: 9898 spectrum library: NIST08.LIB

SI: 83 molecular formula: $C_7H_7NO_2$ CAS: 17747-43-2 mol mass: 137 retention index: 1054

Component name: 3-hydroxypyridine monoacetate $$3-acetoxypyridine $$3-pyridinol, acetate $$

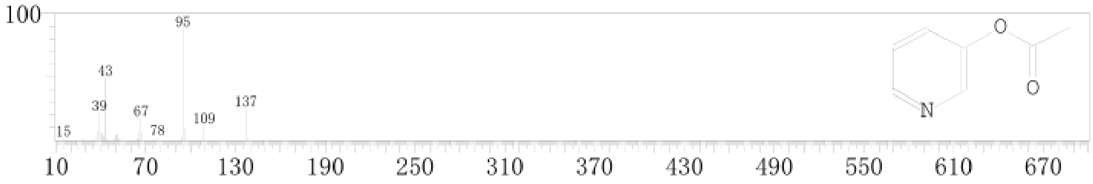

Hit #: 2 input: 9886 spectrum library: NIST08.LIB

SI: 81 molecular formula: $C_7H_7NO_2$ CAS: 14210-20-9 mol mass: 137 retention index: 1054

Component name: 4-pyridinol, acetate (ester) $$4-acetoxypyridine $$4-pyridinyl acetate # $$

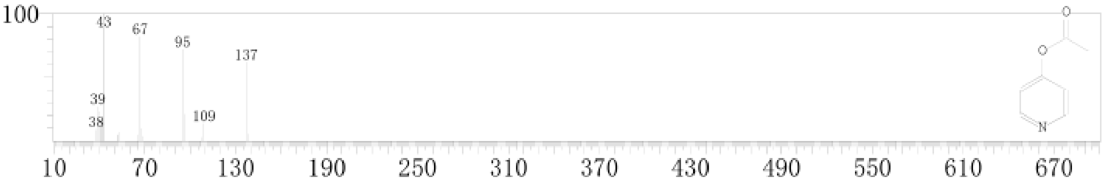

Hit #: 3 enter: 1394 library: NIST08.LIB

SI: 80 molecular formula: C_6H_8O CAS: 62266-35-7 mol mass: 96 retention index: 723

Component name: Ethanone, 1-(methylenecyclopropyl)-1-(2-methylenecyclopropyl) Ethanone $$# $

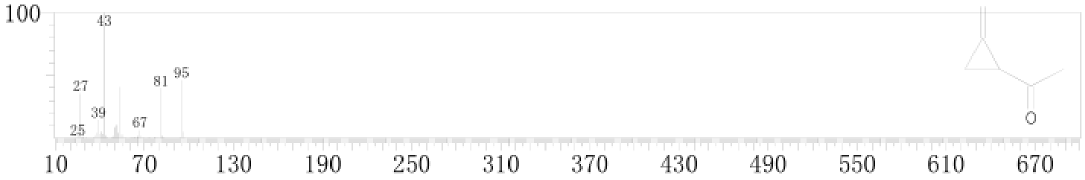

<< target component >>

Line number: 2 retention time: 21.100(scan number: 2053) quality peak: 383 base peak: 43.05(22184)

Original pattern: Averaged 21.033 21.133 (2045-2057) background model: 21.025 (2044).

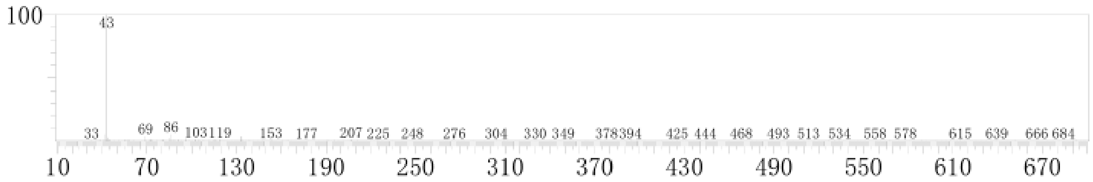

Hit #: 1 input: 4476 spectrum library: NIST08.LIB

SI: 88 molecular formula: $C_5H_8O_3$ CAS: 592-20-1 mol mass: 116 retention index: 822

Component name: 2-propanone, 1-(acetyloxy)- $$2-propanone, 1-hydroxy-, acetate $$Acetol ace

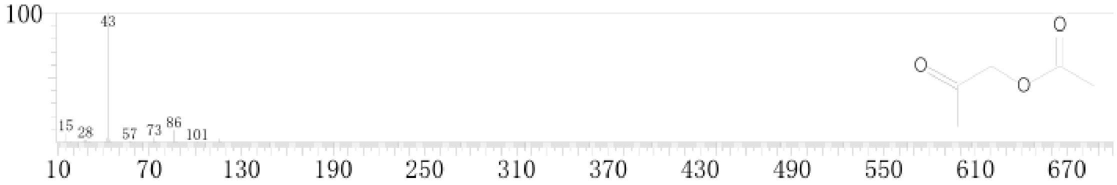

Hit #: 2 input: 803 spectrum library: NIST08.LIB

SI: 88 molecular formula: $C_4H_6O_2$ CAS: 108-05-4 mol mass: 86 retention index: 576

Acetic acid ethenyl ester $$Acetic acid vinyl ester $$Viny

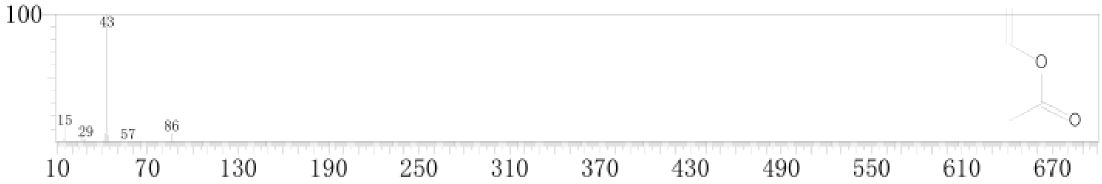

Hit #: 3 input: 4470 library: NIST08.LIB

SI: 87 molecular formula: $C_5H_8O_3$ CAS: 6387-89-9 mol mass: 116 retention index: 776

Component name: 1, 2-propyl acetate $$Oxiranemethanol, acetate $$Acetic acid, oxiran

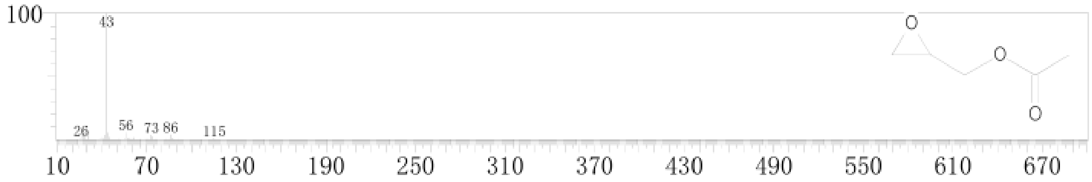

<< target component >>

Line number: 3 retention time: 27.017(number of scans: 2763) mass peak: 364 base peak: 43.05(48237)

Original pattern: Averaged 26.958 27.117 (2756-2775) background model: 27.083 (2771).

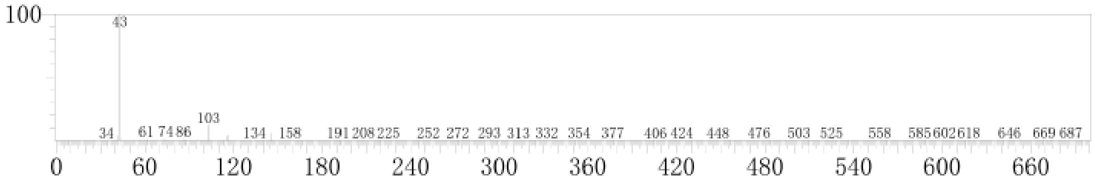

Hit #: 1 input: 54008 spectrum library: NIST08.LIB

SI: 97 molecular formula: $C_9H_{14}O_6$ CAS: 102-76-1 mol mass: 218 retention index: 1354

Component name: Triacetin $$1, 2, 3-propanetriol, triacetate $$Acetin, tri- $$Enzactin $$Fu

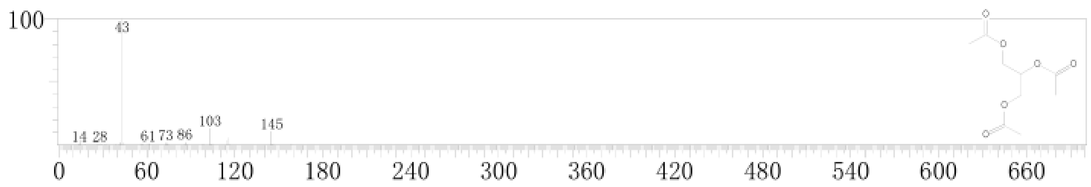

Hit #: 2 input: 28369 spectrum library: NIST08.LIB

SI: 93 molecular formula: $C_7H_{12}O_5$ CAS: 25395-31-7 mol mass: 176 retention index: 1230

Components: 1, 2, 3-propanetriol, diacetate $$Acetin, di-$$Diacetin $$Diacetylglycerol

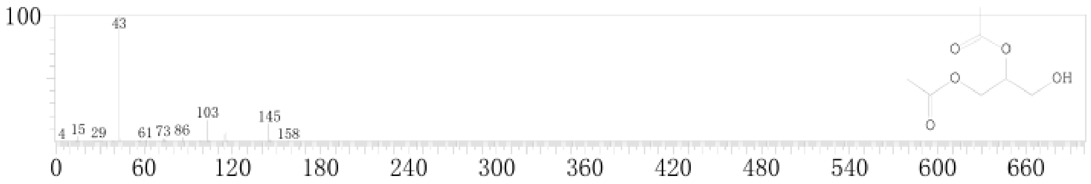

Hit #: 3 input: 44765 spectrum library: NIST08.LIB

SI: 92 molecular formula: $C_8H_{12}O_6$ CAS: 2983-35-9 mol mass: N204 retention index: 1254

Component name: 1, 1, 2-triacetoxyethane $$1, 1, 2-ethanetriol, triacetate $$1, 2-bis (acetyloxy)

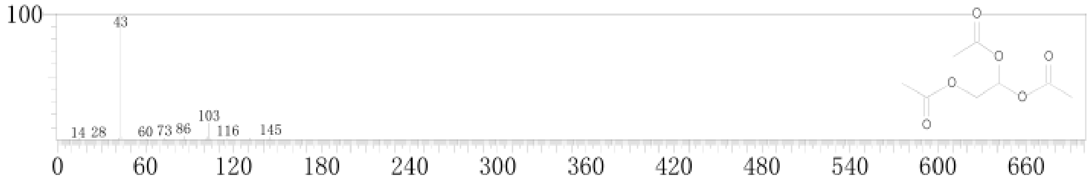

<< target component >>

Line number: 4 retention time: 38.333(number of scans :4121)

Original pattern: Averaged 38.242 38.408 (4110-4130) background model: 38.267 (4113).

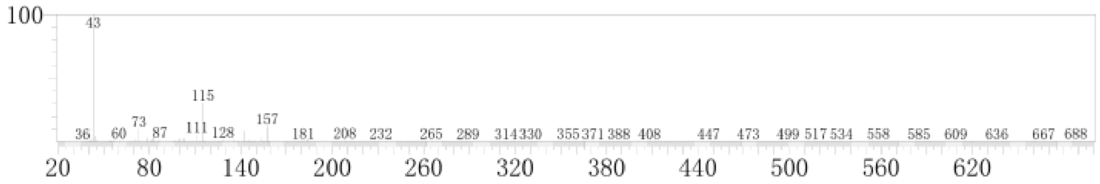

Hit #: 1 input: 133652 spectrum library: NIST08.LIB

SI: 90 molecular formula: $C_{14}H_{20}O_9$ CAS: 27821-11-0 mol mass: 332 retention index: 2081

Component name: alpha.-l-mannopyranose, 6-deoxy-, tetraacetate $$1, 2, 3, 4-tetra -o-acetyl-6-d

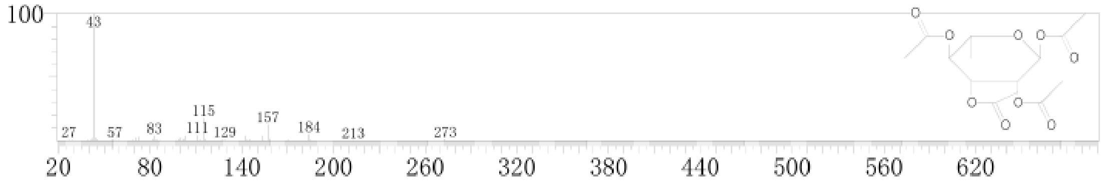

Hit #: 2 input: 133651 spectrum library: NIST08.LIB

SI: 86 molecular formula: $C_{14}H_{20}O_9$ CAS: 0-00-0 mol mass: 332 retention index: 2081

Tetraacetyl. Beta.-d-rhamnose

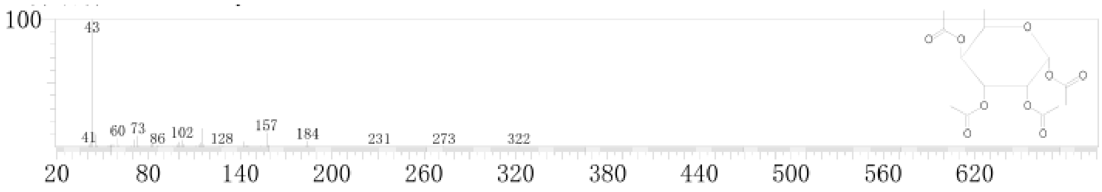

Hit #: 3 input: 133650 spectrum library: NIST08.LIB

SI: 85 molecular formula: $C_{14}H_{20}O_9$ CAS: 0-00-0 mol mass: 332 retention index: 2081

Tetraacetyl. Beta.-l-rhamnose

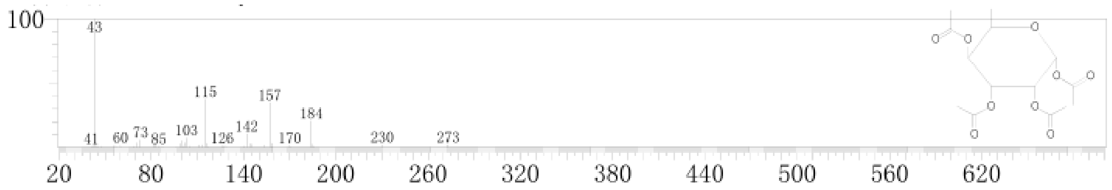

<< target component >>

Line number: 5 retention time: 38.500(number of scans: 4141) mass peak: 359 base peak: 43.05(25045)

Original pattern: Averaged 38.425 38.533 (4132-4145) background model: 38.425 (4132).

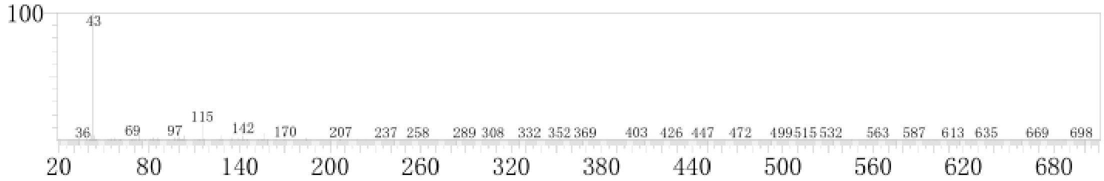

Hit #: 1 input: 133652 spectrum library: NIST08.LIB

SI: 89 molecular formula: $C_{14}H_{20}O_9$ CAS: 27821-11-0 mol mass: 332 retention index: 2081

Component name: alpha.-l-mannopyranose, 6-deoxy-, tetraacetate $$1, 2, 3, 4-tetra -o-acetyl-6-d

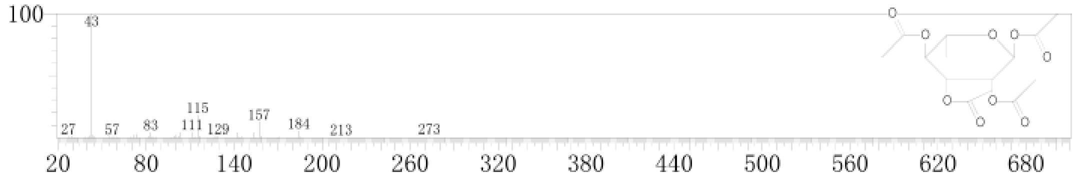

Hit #: 2 input: 124261 library: NIST08.LIB

SI: 87 molecular formula: $C_{13}H_{18}O_9$ CAS: 67226-03-3 mol mass: 318 retention index: 2020

Component name: Lyxopyranose, tetraacetate $$1, 2, 3, 4-tetra-o-acetylpentopyranose # $$

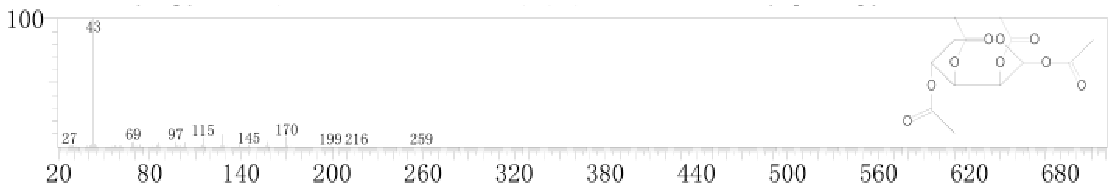

Hit #: 3 input: 135029 spectrum library: NIST08.LIB

SI: 87 molecular formula: $C_{14}H_{22}O_9$ CAS: 0-00-0 molar mass: 334 retention index: 1966

Component name: d-xylitol, 2, 3, 4, 5-tetraacetyl-1-o-methyl -

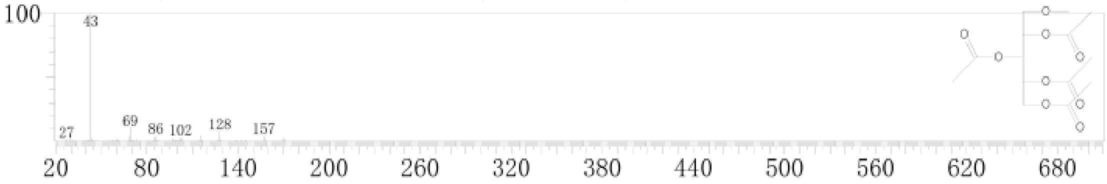

<< target component >>

Line number: 6 retention time: 38.608(scan number: 4154) mass peak: 392 base peak: 43.05(17367)

Original pattern: Averaged 38.583 38.708 (4151-4166) background model: 38.708 (4166).

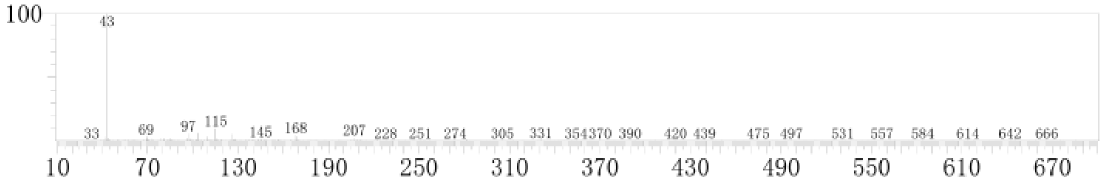

Hit #: 1 input: 122324 spectrum library: NIST08.LIB

SI:86 molecular formula: $C_{13}H_{17}NO_8$ CAS:34360-56-0 molar mass: 315 retention index: 2036

Component name: lyxonitrile, 2, 3, 4, 5-tetraacetate, d-

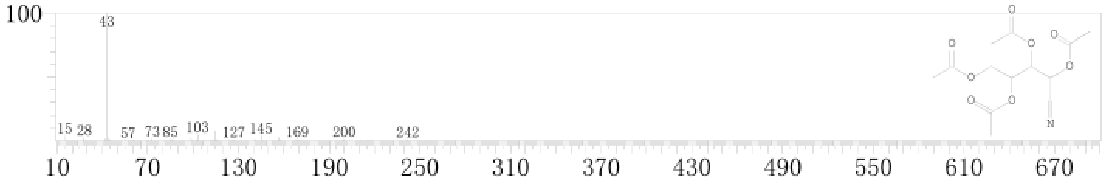

Hit #: 2 input: 164054 spectrum library: NIST08.LIB

SI: 86 molecular formula: $C_{16}H_{22}O_{11}$ CAS: 5531-53-3 mol mass: 390 retention index: 2401

Component name: beta. -d-galactofuranose, pentaacetate $$Galactofuranose, pentaacetate, .bet

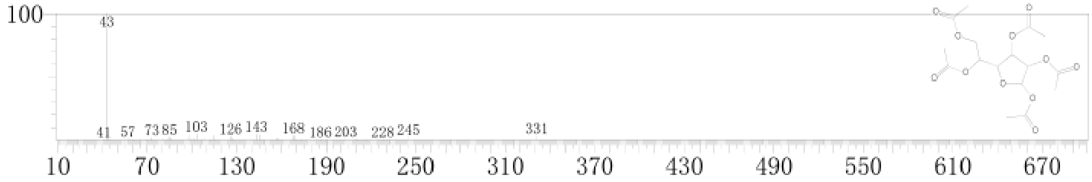

Hit #: 3 input: 170391 spectrum library: NIST08.LIB

SI: 83 molecular formula: $C_{14}H_{19}BrO_9$ CAS: 572-09-8 mol mass: 410 retention index: 2377

Component name: .alpha.-d-glucopyranosyl bromide, tetraacetate $2, 3, 4, 6-tetra -o-acetylhexop

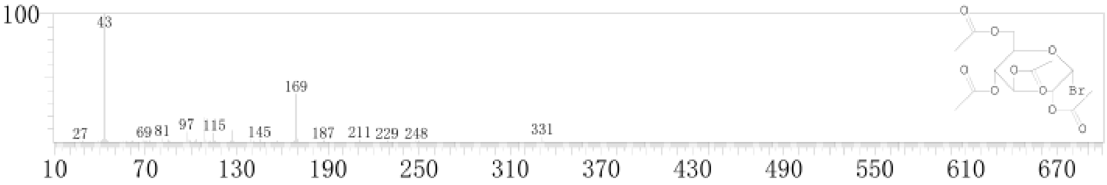

<< target component >>

Line number: 7 retention time: 39.008(scan number: 4202) quality peak: 355 base peak: 43.05(10879)

Original pattern: Averaged 38.942 39.058 (4194-4208) background model: 38.942 (4194).

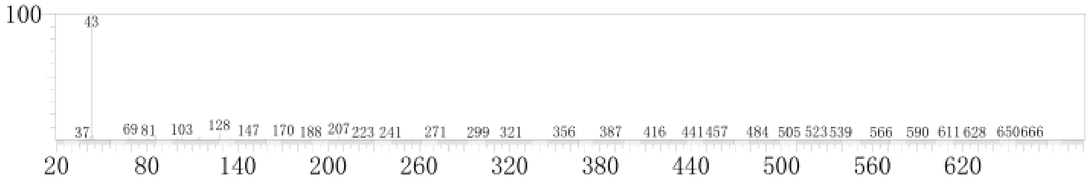

Hit #: 1 input: 124261 library: NIST08.LIB

SI: 87 molecular formula: $C_{13}H_{18}O_9$ CAS: 67226-03-3 mol mass: 318 retention index: 2020

Component name: Lyxopyranose, tetraacetate $$1, 2, 3, 4-tetra-o-acetylpentopyranose # $$

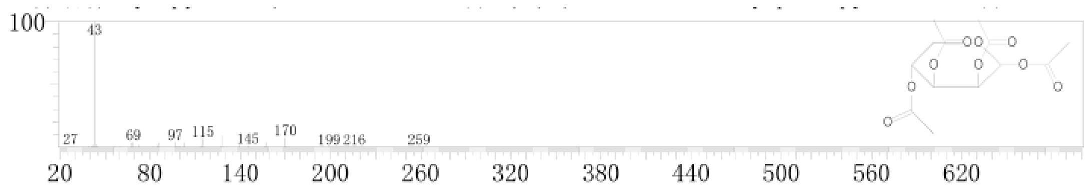

Hit #: 2 input: 135029 spectrum library: NIST08.LIB

SI: 86 molecular formula: $C_{14}H_{22}O_9$ CAS: 0-00-0 molar mass: 334 retention index: 1966

Component name: d-xylitol, 2, 3, 4, 5-tetraacetyl-1-o-methyl -

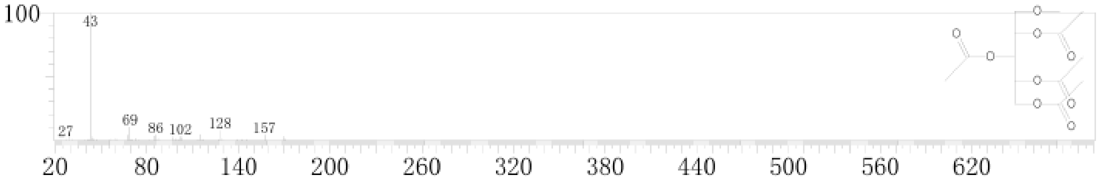

Hit #: 3 input: 93116 spectrum library: NIST08.LIB

SI: 85 molecular formula: $C_{11}H_{14}O_8$ CAS: 0-00-0 mol mass: 274 retention index: 1908

Acetic acid, 4-acetoxy-5-acetoxymethyl 2-oxo-tetrahydro-furan-3-yl ester

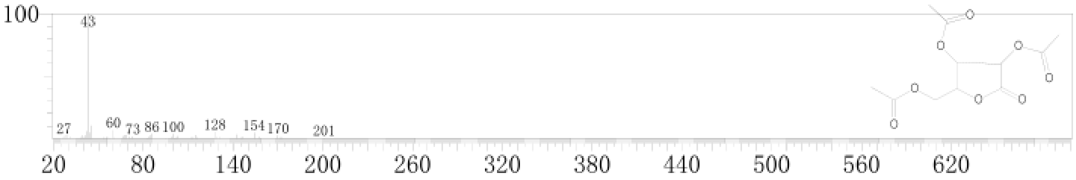

<< target component >>

Line number: 8 retention time: 39.217(number of scans: 4227) quality peak: 414 base peak: 43.05(6496)

Original pattern: Averaged 39.108 39.292 (4214-4236) background model: 39.117 (4215).

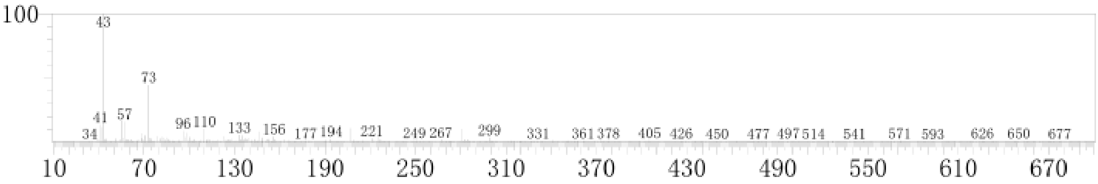

Hit #: 1 input: 8400 spectrum library: NIST08.LIB

SI: 68 molecular formula: $C_6H_{12}O_3$ CAS: 150-96-9 mol mass: 132 retention index: 1066

Component name: 3-hydroxy-3-methylvaleric acid $$3-hydroxy-3-methylpentanoic acid # $$

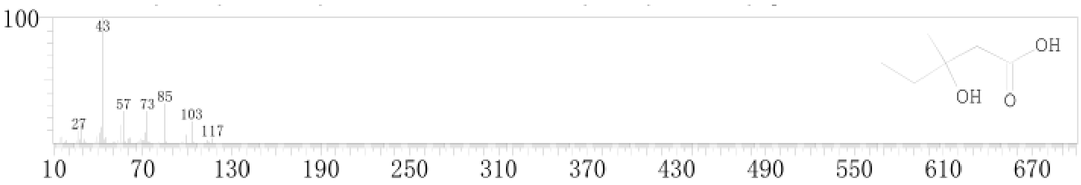

Hit #: 2 input: 7880 library: NIST08.LIB

SI: 68 molecular formula: $C_7H_{14}O_2$ CAS: 107-70-0 molar mass: 130 retention index: 844

Composition name: 2-pentanone, 4-methoxy-4-methyl- $$pent-oxone Solvent $$Pentoxone $$4-meth

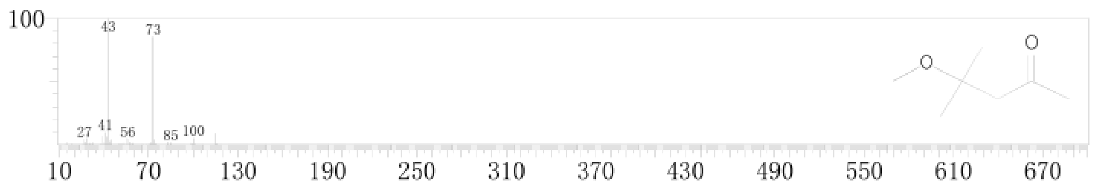

Hit #: 3 input: 18389 spectrum library: NIST08.LIB

SI: 68 molecular formula: $C_{10}H_{20}O$ CAS: 118452-32-7 mol mass: 156 retention index: 1023

Component name: octan-2-one, 3, 6-dimethyl- $$3, 6-dimethyl- 2-octanone # $$$

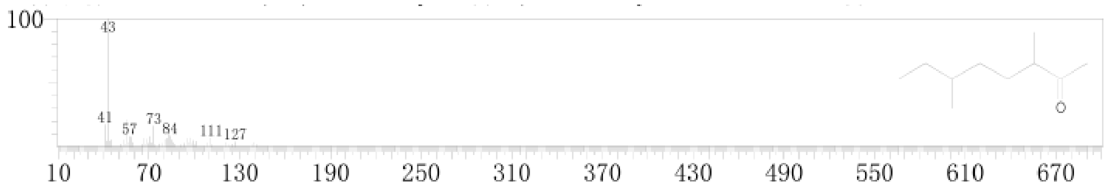

<< target component >>

Line number: 9 retention time: 44.033(number of scans :4805) quality peak: 391 base peak: 43.05(12561)

Original pattern: Averaged 43.892 44.075 (4788-4810) background model: 43.892 (4788).

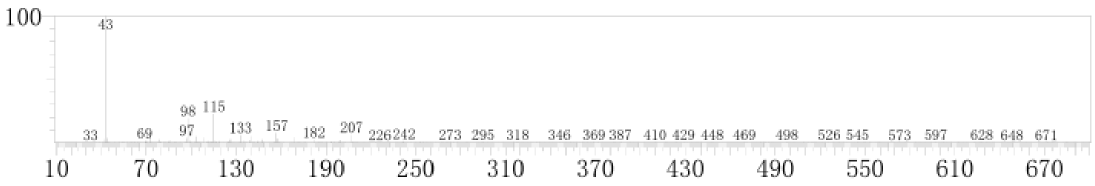

Hit #: 1 input: 164048 spectrum library: NIST08.LIB

SI: 87 molecular formula: $C_{16}H_{22}O_{11}$ CAS: 3891-59-6 mol mass: 390 retention index: 2380

Component name: d-glucose, 2, 3, 4, 5, 6-pentaacetate $$d-glucose pentaacetate $$Glucose pentaa

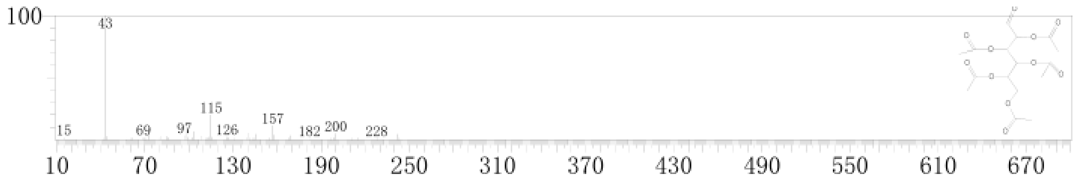

Hit #: 2 input: 164046 spectrum library: NIST08.LIB

SI: 87 molecular formula: $C_{16}H_{22}O_{11}$ CAS:604-68-2 mol mass: 390 retention index: 2447

Glucopyranose, pentaacetate, .alpha. -d-glucopyranose, pentaacetate $$Glucopyranose, pentaacetate, .alpha.

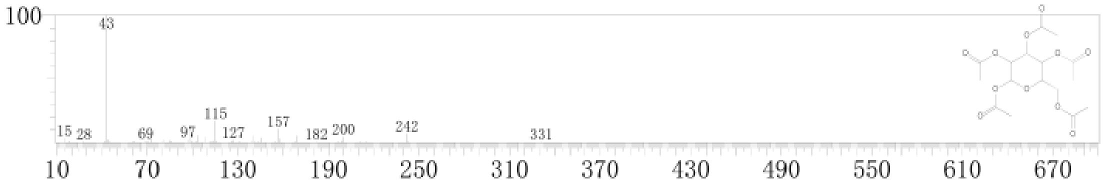

Hit #: 3 input: 143550 library: NIST08.LIB

SI: 85 molecular formula: $C_{14}H_{20}O_{10}$ CAS: 0-00-0 mol mass: 348 retention index: 2307

Component name: 1, 3, 4, 6-tetra -o-acetyl -. Beta.-d-glucopyranose

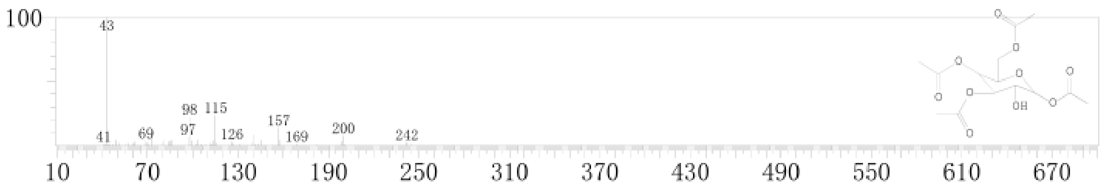

<< target component >>

Line number: 10 retention time: 44.167(number of scans: 4821) quality peak: 396 base peak: 43.05(44988)

Original pattern: Averaged 44.092 44.208 (4812-4826) background model: 44.100 (4813).

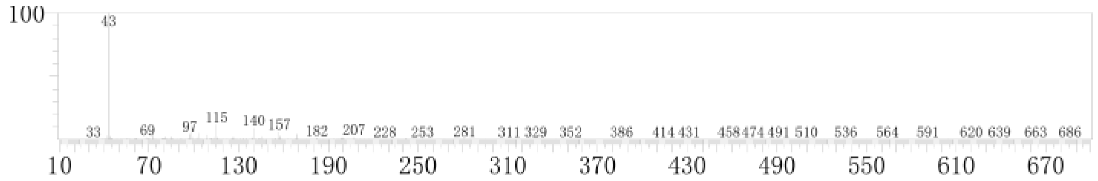

Hit #: 1 input: 164046 spectrum library: NIST08.LIB

SI: 93 molecular formula: $C_{16}H_{22}O_{11}$ CAS: 604-68-2 mol mass: 390 retention index: 2447

Glucopyranose, pentaacetate, .alpha. -d-glucopyranose, pentaacetate $$Glucopyranose, pentaacetate, .alpha.

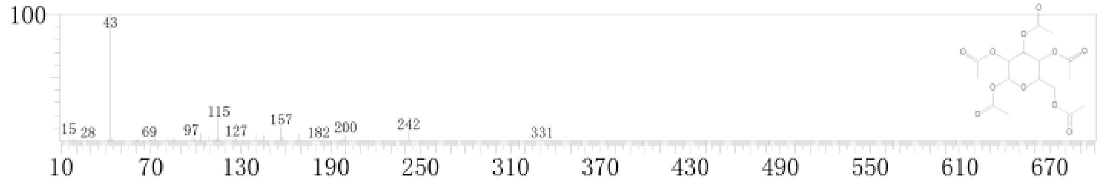

Hit #: 2 input: 164048 spectrum library: NIST08.LIB

SI: 92 molecular formula: $C_{16}H_{22}O_{11}$ CAS: 3891-59-6 mol mass: 390 retention index: 2380

Component name: d-glucose, 2, 3, 4, 5, 6-pentaacetate $$d-glucose pentaacetate $$Glucose pentaa

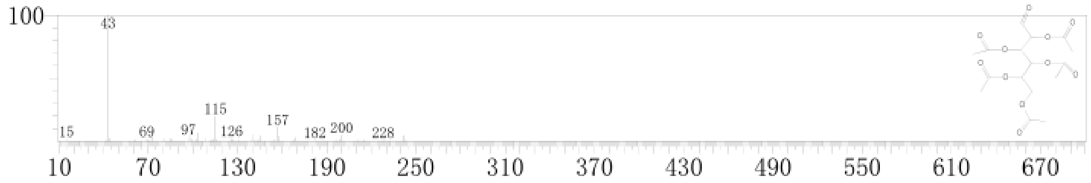

Hit #: 3 input: 164045 spectrum library: NIST08.LIB

SI: 91 molecular formula: $C_{16}H_{22}O_{11}$ CAS: 0-00-0 mol mass: 390 retention index: 2447

Component name: .alpha.-d-galactose pentaacetate

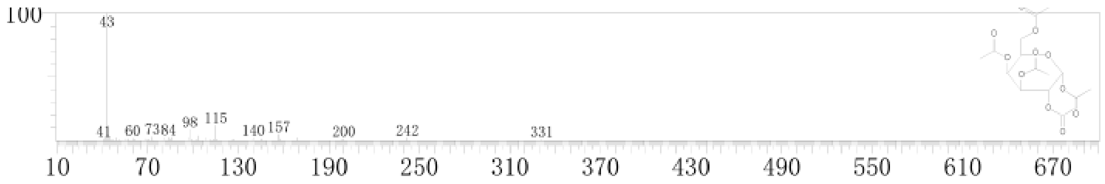

<< target component >>

Line number: 11 retention time: 44.242(number of scans: 4830) quality peak: 386 base peak: 43.05(17126)

Original pattern: Averaged 44.208 44.283 (4826-4835) background model: 44.283 (4835).

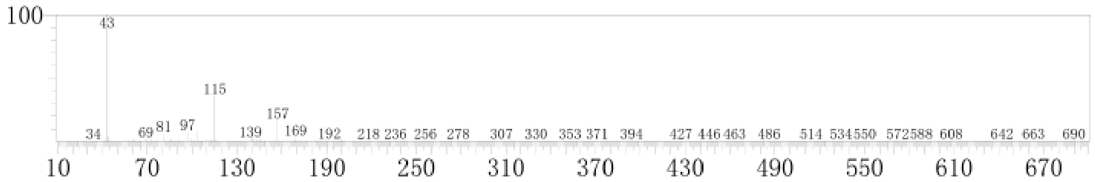

Hit #: 1 input: 164048 spectrum library: NIST08.LIB

SI: 85 molecular formula: $C_{16}H_{22}O_{11}$ CAS: 3891-59-6 mol mass: 390 retention index: 2380

Component name: d-glucose, 2, 3, 4, 5, 6-pentaacetate $$d-glucose pentaacetate $$Glucose pentaa

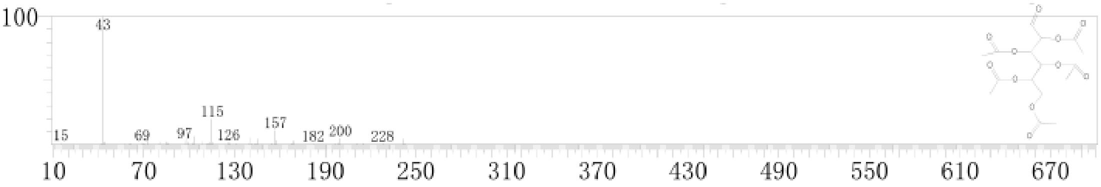

Hit #: 2 input: 164046 spectrum library: NIST08.LIB

SI: 84 molecular formula: $C_{16}H_{22}O_{11}$ CAS: 604-68-2 mol mass: 390 retention index: 2447

Glucopyranose, pentaacetate,. alpha. -d-glucopyranose, pentaacetate $$Glucopyranose, pentaacetate,.alpha.

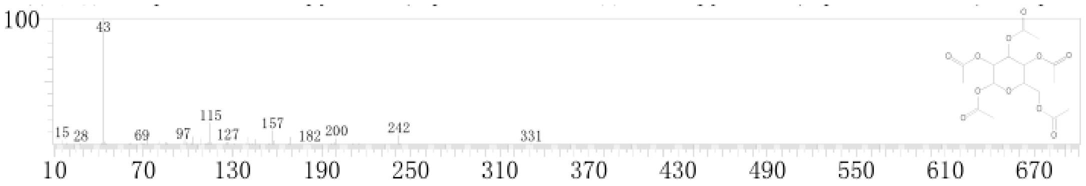

Hit #: 3 input: 176111 spectrum library: NIST08.LIB

SI: 84 molecular formula: $C_{19}H_{30}O_{11}$ CAS: 0-00-0 molar mass: 434 retention index: 2502

Component name: d-mannitol, 2,3,4,5, 6-pentaacetyl-1-o-isopropyl -

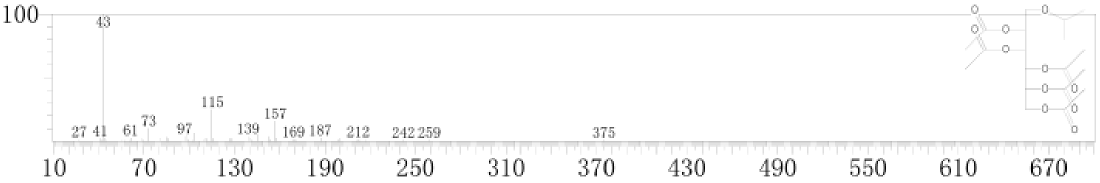

<< target component >>

Line number: 12 retention time: 44.350(number of scans: 4843) quality peak: 418 base peak: 43.05(39612)

Original pattern: Averaged 44.292 44.417 (4836-4851) background model: 44.283 (4835).

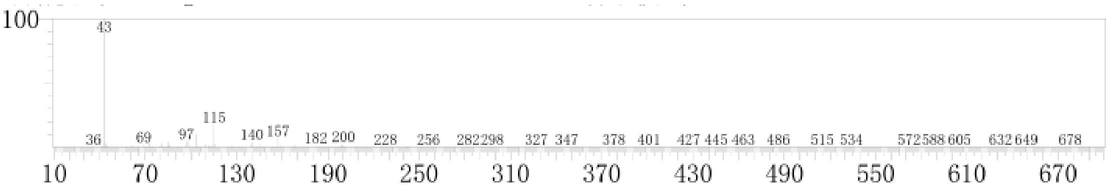

Hit #: 1 input: 164048 spectrum library: NIST08.LIB

SI: 92 molecular formula: $C_{16}H_{22}O_{11}$ CAS: 3891-59-6 mol mass: 390 retention index: 2380

Component name: d-glucose, 2, 3, 4, 5, 6-pentaacetate $$d-glucose pentaacetate $$Glucose pentaa

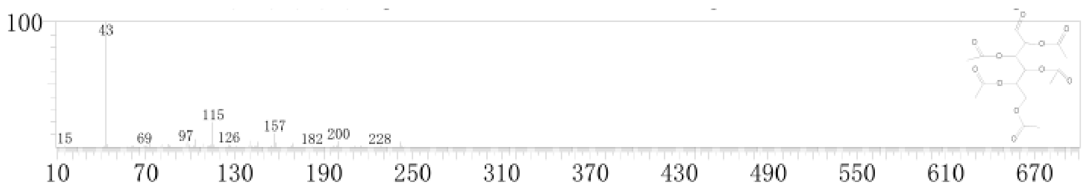

Hit #: 2 input: 164046 spectrum library: NIST08.LIB

SI: 92 molecular formula: $C_{16}H_{22}O_{11}$ CAS: 604-68-2 mol mass: 390 retention index: 2447

Glucopyranose, pentaacetate, .alpha. -d-glucopyranose, pentaacetate $$Glucopyranose, pentaacetate, .alpha.

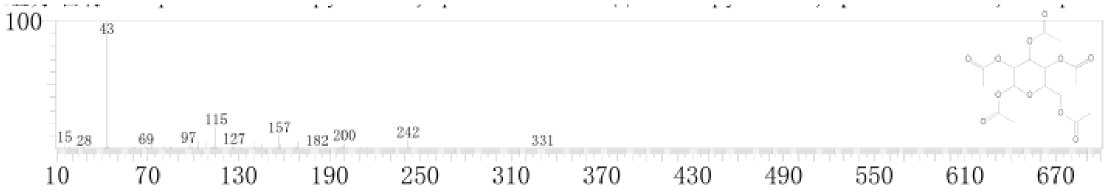

Hit #: 3 input: 164045 spectrum library: NIST08.LIB

SI: 90 molecular formula: $C_{16}H_{22}O_{11}$ CAS: 0-00-0 mol mass: 390 retention index: 2447

Component name:. alpha. -d-galactose pentaacetate

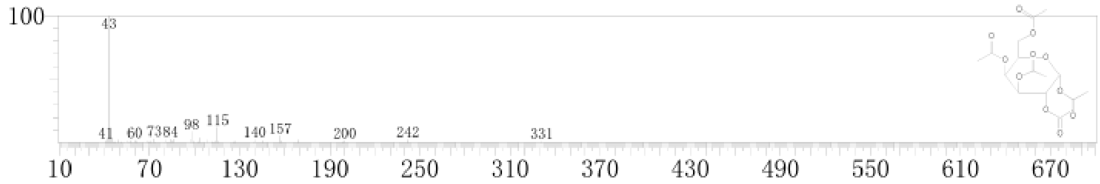

Supplement: Supplemental Information 1 [file peerj-08-9077-s001.zip › original GC-MS data/Translation version-----AHP-M-2.pdf]
